# A single-cell atlas reveals shared and distinct immune responses and metabolic profiles in SARS-CoV-2 and HIV-1 infections

**DOI:** 10.3389/fgene.2023.1105673

**Published:** 2023-03-13

**Authors:** Tony Pan, Guoshuai Cao, Erting Tang, Yu Zhao, Pablo Penaloza-MacMaster, Yun Fang, Jun Huang

**Affiliations:** ^1^ Pritzker School of Molecular Engineering, University of Chicago, Chicago, IL, United States; ^2^ Department of Microbiology-Immunology, Northwestern University, Chicago, IL, United States; ^3^ Biological Sciences Division, University of Chicago, Chicago, IL, United States

**Keywords:** HIV, Single-cell RNA sequencing, SARS-CoV-2, type 1 interferon, inflammation, metabolic signaling

## Abstract

**Introduction:** Within the inflammatory immune response to viral infection, the distribution and cell type-specific profiles of immune cell populations and the immune-mediated viral clearance pathways vary according to the specific virus. Uncovering the immunological similarities and differences between viral infections is critical to understanding disease progression and developing effective vaccines and therapies. Insight into COVID-19 disease progression has been bolstered by the integration of single-cell (sc)RNA-seq data from COVID-19 patients with data from related viruses to compare immune responses. Expanding this concept, we propose that a high-resolution, systematic comparison between immune cells from SARS-CoV-2 infection and an inflammatory infectious disease with a different pathophysiology will provide a more comprehensive picture of the viral clearance pathways that underscore immunological and clinical differences between infections.

**Methods:** Using a novel consensus single-cell annotation method, we integrate previously published scRNA-seq data from 111,566 single PBMCs from 7 COVID-19, 10 HIV-1^+^, and 3 healthy patients into a unified cellular atlas. We compare in detail the phenotypic features and regulatory pathways in the major immune cell clusters.

**Results:** While immune cells in both COVID-19 and HIV-1^+^ cohorts show shared inflammation and disrupted mitochondrial function, COVID-19 patients exhibit stronger humoral immunity, broader IFN-I signaling, elevated Rho GTPase and mTOR pathway activity, and downregulated mitophagy.

**Discussion:** Our results indicate that differential IFN-I signaling regulates the distinct immune responses in the two diseases, revealing insight into fundamental disease biology and potential therapeutic candidates.

## Introduction

Viral infection in humans initiates a coordinated response between the innate and adaptive immune systems. This defense response involves: recruitment and activation of inflammatory cell populations, such as macrophages and monocytes (Koyama et al., 2008); IFN-I signaling, which drives transcription of multifunctional IFN-stimulated effector molecules (Koyama et al., 2008; McNab et al., 2015); and significant metabolic shifts in immune cells, attributed to increased cytokine signaling (Chandler et al., 2016; Sumbria et al., 2020). Cytotoxic T cells clear infected cells *via* cytokine-mediated destruction or direct killing, while helper T cells prime B cells to produce antibodies, which neutralize viral replication. However, the distribution and cell type-specific profiles of the different immune cell populations vary across different viruses/diseases, conditions, and stages of disease progression (MacParland et al., 2018; Travaglini et al., 2020; Delorey et al., 2021). Within what seems like a common inflammatory program, the immune-mediated pathways are virus-specific.

Single-cell RNA sequencing (scRNA-seq), which can accurately annotate individual cells, is widely used to characterize heterogeneity within immune cell subsets (Tang et al., 2009; Treutlein et al., 2014; Gawad et al., 2016; Reyfman et al., 2019; Chow et al., 2021). Integration of scRNA-seq data to compare immune responses across different viral diseases (typically with similar pathophysiologies) can reveal similarities and differences in the inflammatory immune response. This strategy has drawn increased interest since the onset of the COVID-19 pandemic as it can facilitate translation of insights from one disease to another. For example, Lee et al., used scRNA-seq to examine peripheral blood mononuclear cells (PBMCs) from patients with influenza or severe COVID-19, reporting a common enrichment of inflammatory monocytes upregulating TNF-α, IL-1β, and IFN-I, alongside influenza-specific expression of *STAT1* and *TLR4* and COVID-19-specific expression of *NFKB1/2* and *STAT4* (Lee et al., 2020). Schuurman, Reijnders, et al. found that monocytes and NK cells from patients with SARS-CoV-2-derived community-acquired pneumonia (CAP) expressed higher levels of interferon-stimulated genes (ISGs) compared to monocytes and NK cells from patients with non-SARS-CoV-2-derived CAP (Schuurman et al., 2021). The COMBAT Consortium generated an integrated blood atlas of COVID-19, influenza, and sepsis which revealed a shared neutrophil signature, alongside elevated plasmablast frequencies, type-2 T cell responses, and plasma concentrations of inflammatory cytokines (such as IL-6 and IL-8) in patients with COVID-19 (COvid-19 Multi-omics Blood ATlas (COMBAT) Consortium, 2022). Altogether, these studies point to a common theme of inflammation regulated by specific genes and cytokines, particularly IFN-I. However, the myriad virus-specific pathways activated by the immune system cannot be revealed by comparing only related diseases. We propose that a high-resolution, systematic comparison between immune cells from SARS-CoV-2 infection and an inflammatory infectious disease with a different pathophysiology will provide a more comprehensive picture of viral clearance pathways.

SARS-CoV-2 and HIV-1 are RNA viruses and thus exhibit high mutation rates relative to DNA viruses. SARS-CoV-2 and HIV-1 are both highly virulent, but disease progression differs substantially. Immune cell subsets such as macrophages and monocytes have been implicated in driving inflammatory cytokine signaling during both SARS-CoV-2 and HIV-1 infection. (Deeks et al., 2013; Campbell et al., 2014; Schulte-Schrepping et al., 2020; Knoll et al., 2021). However, most of the mortality and morbidity observed with SARS-CoV-2 infection occurs within days of infection, compared to months or years with HIV-1 infection. Furthermore, neutralizing antibody responses are rapidly generated following SARS-CoV-2 infection, but these take many years to develop in people living with HIV-1 (Stamatatos et al., 2009; Cotugno et al., 2021; Dangi et al., 2021). These clinical and immunological differences are driven in part by how the host responds to distinct viral infections.

Here, we sought to identify the disease-specific drivers and mediators of inflammation, IFN-I signaling, and metabolism pathways of immune-mediated viral clearance in patients with COVID-19 and HIV-1. We present a comprehensive strategy to integrate scRNA-seq data of 111,566 single PBMCs from 7 COVID-19, 10 HIV-1^+^, and 3 healthy patients from previously published datasets (Wilk et al., 2020; Kazer et al., 2020; Wang et al., 2020; 10xGenomics, 2020). Our strategy combines the advantages of manual annotation, correlation-based label transfer and deep-learning-based classification to generate a high-quality unified cellular atlas of the immune landscape. We compare in detail the phenotypic features and regulatory pathways in each of the major immune compartments (T cells, B cells, natural killer cells, dendritic cells, and monocytes). We find common signatures of inflammation and disrupted mitochondrial function in both COVID-19 and HIV-1. Moreover, we identify important differences in cell signaling, antibody diversity, IFN-I signaling, and metabolic function, including differential IFN-I signaling that likely regulates the distinct immune responses against the two diseases.

## Materials and methods

### Preprocessing, integration, and clustering

Raw single-cell count matrices were collected from publicly available sources (Tables 1–3) (Kazer et al., 2020; Wang et al., 2020; Wilk et al., 2020; 10xGenomics, 2020) and merged. We performed quality control and downstream analysis using the Seurat package (v4.0.4) (Stuart et al., 2019). We removed cells with greater than 15,000 unique molecular modifiers (UMIs) or fewer than 500 UMIs, as well as greater than 20% mitochondrial reads per cell. We performed log-based normalization with the “NormalizeData” function with the “LogNormalize” parameter and selected the top 10,000 variable features with the “vst” parameter using “FindVariableFeatures”. We scaled and centered the count matrix using the “ScaleData” function and supplied “percent.mito” as a latent variable to regress out the effect of percentage mitochondrial reads. We performed principal component analysis (PCA) on the top 100 PCs using the “RunPCA” function. To remove study-specific batch effects, we performed integration across each patient using the Harmony algorithm (v0.1.0) (Korsunsky et al., 2019) on the top 50 principal components (PCs) with the “RunHarmony” function. We then performed Uniform Manifold Approximation and Projection (UMAP) reduction using the “RunUMAP” function on the top 50 PCs with “min.dist” = 0.1 and “n.neighbors” = 20. We ran the “FindNeighbors” function on the top 50 Harmony dimensions, then performed Louvain clustering using the “FindClusters” function with a resolution of 0.3. We removed doublets using the scDblFinder package (v1.10.0) (McGinnis et al., 2019) by supplying sample source, 50 Harmony dimensions, 10,000 variable features, and 100 PCs as parameters. We annotated the clusters using known cell type-specific markers, resulting in a total of 19 cell types, including 7 CD8^+^ T cell subtypes, 3 monocyte subtypes, and 4 CD4^+^ T cell subtypes, and added the labels to the main object.

**TABLE 1:** Characteristics of selected data from COVID-19 patients from Wilk et al.

ID	Condition	Age	Sex	Ventilated?	Clinical outcome
C1	Severe COVID-19	60–69	M	No/Yes	Discharged to rehab on room air
C2	Severe COVID-19	40–49	M	No	Discharged home
C3	Severe COVID-19	30–39	M	Yes	Tracheostomy, prolonged ICU and hospital course
C4	Severe COVID-19	30–39	M	Yes	Discharged home
C5	Severe COVID-19	50–59	M	No	Discharged home
C6	Severe COVID-19	>80	M	Yes	Deceased
C7	Severe COVID-19	20–29	M	No	Discharged home

**TABLE 2:** Characteristics of selected data from HIV + individuals from Kazer et al.

ID	Age	Sex	Ethnicity	Controller?	Weeks post infection at sampling point(s)	ART suppression
P1	24	F	African	No	0, 1, 2, 3, 4, 26, 52	None
P2	21	F	African	No	0, 1, 2, 3, 4, 26, 52	None
P3	24	F	African	Yes	0, 1, 2, 3, 4, 26, 52	None
P4	21	F	African	Yes	0, 1, 2, 3, 4, 26, 52	None

**TABLE 3:** Characteristics of selected data from HIV + individuals from Wang et al.

ID	Age	Sex	Ethnicity	Controller?	Duration of infection (years)	ART suppression status	Plasma HIV RNA (copies/mL)
Q1	59	Male	Non-Hispanic	No	0.3	None	585,100
Q2	56	Male	African American	No	27	Intermittent	185,072
Q3	33	Male	White	No	7.6	Full	<20
Q4	58	Male	Other	No	22	Full	<20
Q5	36	Male	African American	No	3.5	None	259,111
Q7	60	Male	Other	No	11	Full	<20

### Cell subset annotation

For manual annotation, we subsetted the three major cell populations (T cells + NK cells, B cells + Plasmablasts, and Dendritic cells + Monocytes) and separately performed normalization, scaling, feature selection, PCA, integration, UMAP, and clustering. For reference-based annotation, we utilized the “SingleR” method from the SingleR package (v1.4.1) (Aran et al., 2019) using data from (Monaco et al., 2019) and default parameters and transferred the fine and coarse labels to the main object. In total, we found 7 major cell types and 27 subtypes with SingleR. For deep learning-based annotation, we used the scANVI package (v0.7.0) from the scvi-tools library (Xu et al., 2021) to train a deep generative model using reference data from (Ren et al., 2021). We first merged the raw counts from our object data with raw counts from the reference into a combined AnnData object. We normalized and logarithmized the matrix with the Scanpy package (Wolf et al., 2018) (v1.4.5) using the “normalize_total” method with “target_sum” = 10,000 and “log1p” method. We found highly variable genes using the “highly_variable_genes” method with “flavor” = “seurat_v3” and “n_top_genes” = 4000. To improve the accuracy of the model, we performed hierarchical clustering on the reference data and merged labels that fell under a common hierarchy, resulting in 32 total labels: 5 B cell subsets, 3 DC subsets, 4 monocyte subsets, 7 CD4^+^ T cell subsets, and 8 CD8^+^ T cell subsets (Supplementary Figures S1A, S1B). We applied the resulting labels to the reference data. We subsampled approximately 500 cells from each cell subset from the reference data to act as the training set and built the model with a latent dimensionality of 30 and 2 hidden layers using the “model.SCANVI” method. We then trained the model using 300 passes for semi-supervised training using the “train” method. We obtained the labels using the “predict” method and transferred the labels to the main object. Our resulting model had an overall accuracy of 76% and a median F1 score of .78, which was higher than the reported accuracy of existing methods such as LAmDA, scmapcluster, and LDA (Abdelaal et al., 2019), in addition to a high purity score, which was computed using the ROGUE package (v1.0) (Liu et al., 2020) (Supplementary Figures S1C–S1E). The labels were further merged based on similarity of marker gene expression. Two clusters (proliferating monocyte and platelet + monocyte) were found to have high doublet content and were removed, resulting in 25 final labels.

### Consensus annotation

Generation of consensus markers was performed using the following steps:

1. Compare manual and SingleR labels. If labels are identical, leave the label as-is.
2. If one label is at higher resolution (i.e. is a subset of the other), assign the higher resolution label.
3. If the two labels are inconsistent, subset out and pool with similarly inconsistent labels. Plot gene expression using markers of either label type. Assign the label with corresponding marker expression (Consensus 1).
4. Repeat 1-3 using Consensus 1 and scANVI labels.

**FIGURE 1:**
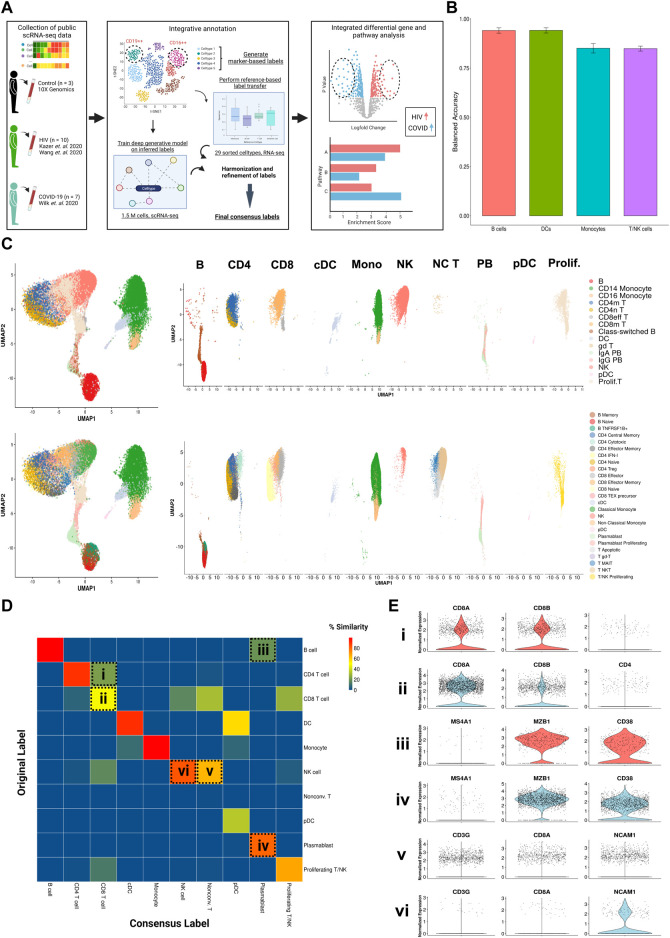
Consensus clustering method to annotate single cell transcriptomic data from multiple sources. **(A)** Illustrated workflow of data collection, consensus annotation, and downstream analysis. **(B)** Balanced accuracy of trained scANVI model on cell labels derived from Ren Cell 2021. Error bars denote variation of accuracy across labels within major cell categories. **(C)** Left: Uniform manifold approximation and projection (UMAP) embeddings of the integrated datasets colored by original (top) and consensus labels (bottom). Right: UMAP embeddings split by major cell categories colored by original (top) and consensus labels (bottom) illustrating the contrast in cell proportions using consensus method. **(D)** Confusion matrix illustrating percentage overlap of original labels and consensus labels across major cell categories. Percentage overlap was calculated by dividing each cell count by the total number of cells in each column. **(E)** Violin plots of canonical normalized gene expression of designated cell populations indicated in 1D (rows).

In the T cell compartment, a significant proportion of CD8^+^ T cells were originally classified as CD4^+^ (Figure 1D ‘i, ii’). When comparing the expression of canonical genes *CD8A*, *CD8B*, and *CD4* in this population (Figure 1E ‘i’) to the expression of the main cluster of CD8^+^ T cells (Figure 1E ‘ii’), we saw that levels were markedly similar, leading us to conclude that they are indeed CD8^+^ T cells. Similarly, we used expressions of *MZ4A1* (a canonical B cell marker), *MZB1*, and *CD38* (canonical plasmablast markers, Figure 1E ‘iii, iv’) to confirm that the population indicated in Figure 1E ‘iii’ are plasmablasts instead of B cells, and the expression of *CD3G*, *CD8A*, and *NCAM1* (a canonical NK cell marker, Figure 1E ‘v, vi’) to confirm that the population indicated in Figure 1E ‘v’ are unconventional T cells instead of NK cells.

### Cell type composition comparison

**FIGURE 2:**
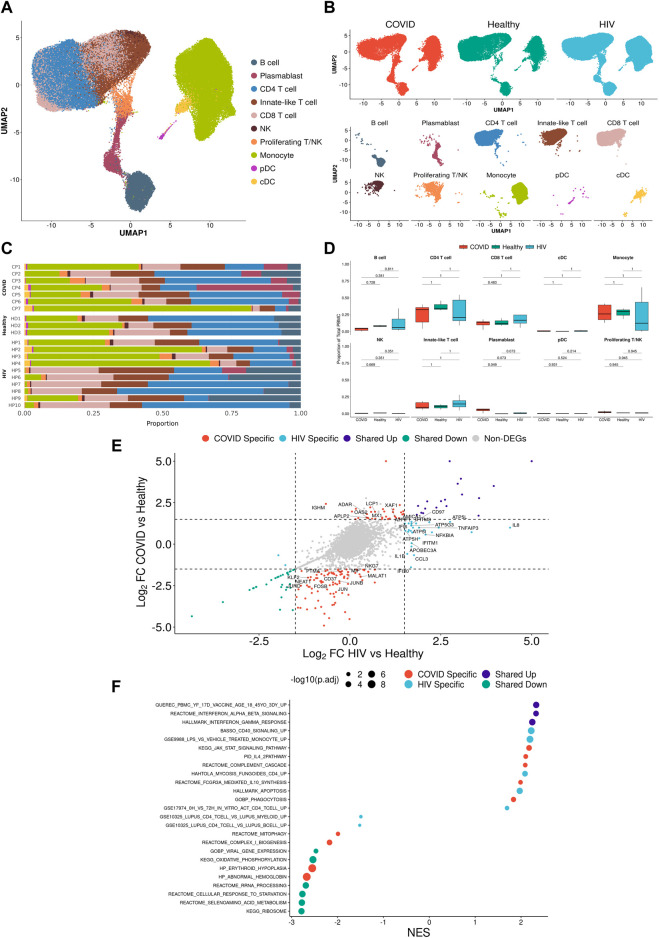
Integrated single-cell landscape of PBMC in HIV-1, COVID-19, and healthy controls. **(A)** UMAP embeddings of integrated HIV-1^+^ and COVID-19 patients together with healthy controls colored by major cell populations. **(B)** Top: UMAP split across disease conditions after regressing out patient-specific effects using the Harmony algorithm. Bottom: UMAP highlighting distribution of major cell populations. **(C)** Stacked bar plots of the relative frequency of major cell populations present in each patient. CP: COVID-19 patient. HP: HIV-1^+^ patient. HD: Healthy donor.**(D)** Box plots of the proportions of each PBMC subset across each disease condition. Proportions are computed for each patient by dividing their number of cells in each subset by their total number of PBMCs. *p*-values are computed with Wilcoxon signed-rank test with Holm-Bonferroni adjustment.**(E)** Double differential gene expression plot of genes that are differentially expressed between COVID-19 patients compared to healthy controls (*Y*-axis) or differentially expressed between HIV-1^+^ patients compared to healthy controls (*X*-axis). Log2FC: Log-2-fold change. **(F)** Dot plot of enriched biological pathways from significantly differentially expressed genes (*p* < .05) that were found to be upregulated (right, positive) or downregulated (left, negative) compared to healthy controls. Size of dot corresponds to adjusted *p*-value of enriched pathway. NES: Normalized enrichment score.

**FIGURE 3:**
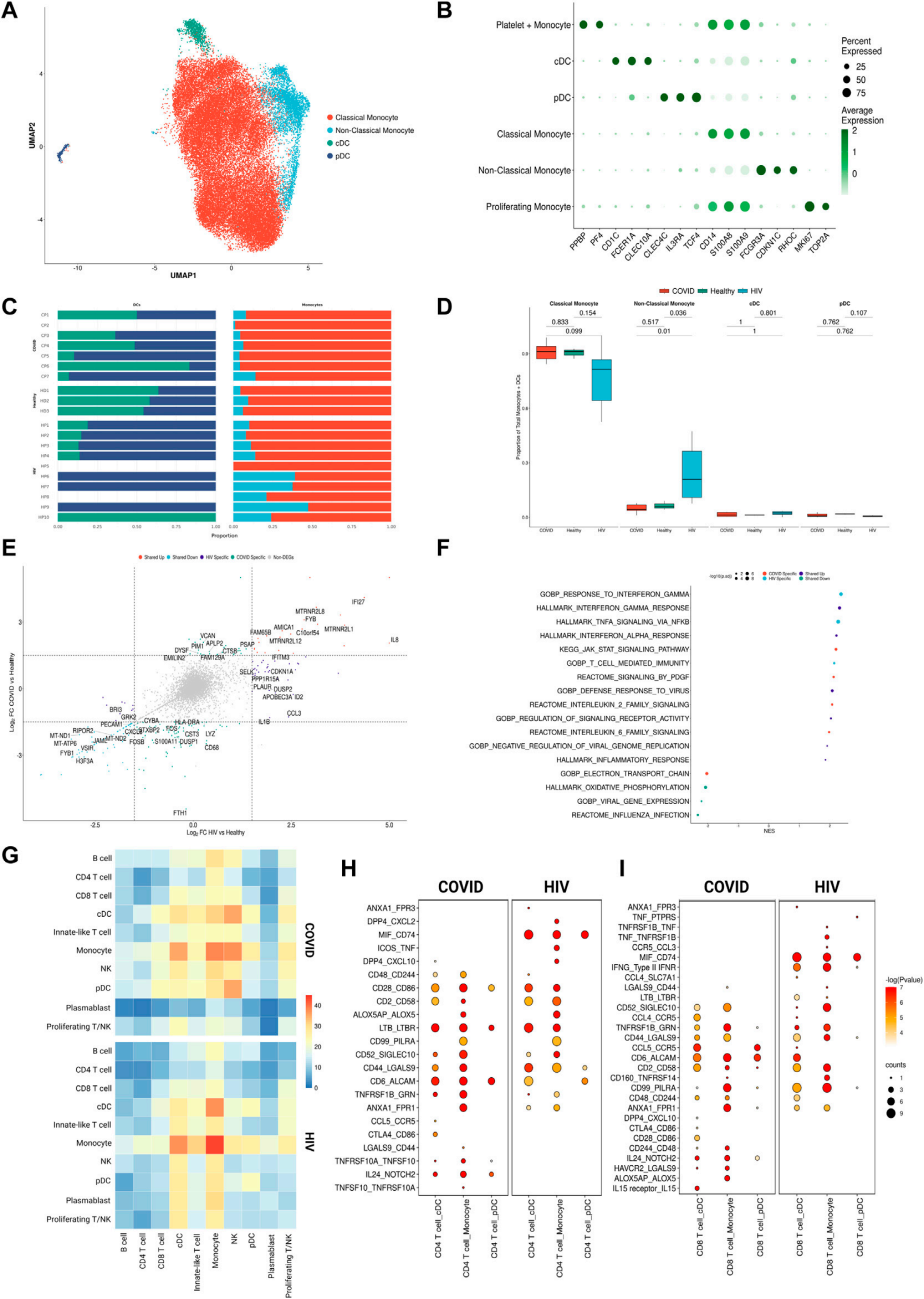
Monocytes in COVID-19 and HIV-1 share inflammatory signatures. **(A)** UMAP embeddings of monocytes and DCs colored by subtype. **(B)** Dot plot of canonical monocyte and DC marker expression across subtypes. **(C)** Stacked bar plots of the relative frequency of subtypes present in each patient. **(D)**Box plots of the proportions of each myeloid subset across each disease condition. Proportions are computed for each patient by dividing their number of cells in each DC/monocyte subset by their total number of DCs + monocytes. *p*-values are computed with Wilcoxon signed-rank test with Holm-Bonferroni adjustment. **(E)** Double differential gene expression plot of genes that are differentially expressed between COVID-19 patients compared to healthy controls or differentially expressed between HIV-1^+^ patients compared to healthy controls. **(F)** Dot plot of enriched biological pathways from differentially expressed genes that were found to be upregulated (right, positive) or downregulated (left, negative) compared to healthy controls. **(G)** Heatmap of the number of receptor-ligand interactions between each cell type in COVID-19 patients (top) and HIV-1^+^ patients (bottom). **(H)** Dot plot of selected receptor-ligand interactions between CD4^+^ T cells and monocytes/DCs in COVID-19 patients (left) versus HIV-1^+^ patients (right). Color of each dot corresponds to the inverse log of the *p*-value of the interaction. Size of the dot corresponds to the number of patients the interaction was found to be significant in. **(I)** Dot plot of selected receptor-ligand interactions between CD8^+^ T cells and monocytes/DCs in COVID-19 patients (left) versus HIV-1^+^ patients (right). Color of each dot corresponds to the inverse log of the *p*-value of the interaction. Size of the dot corresponds to the number of patients for which the interaction was found to be significant.

**FIGURE 4:**
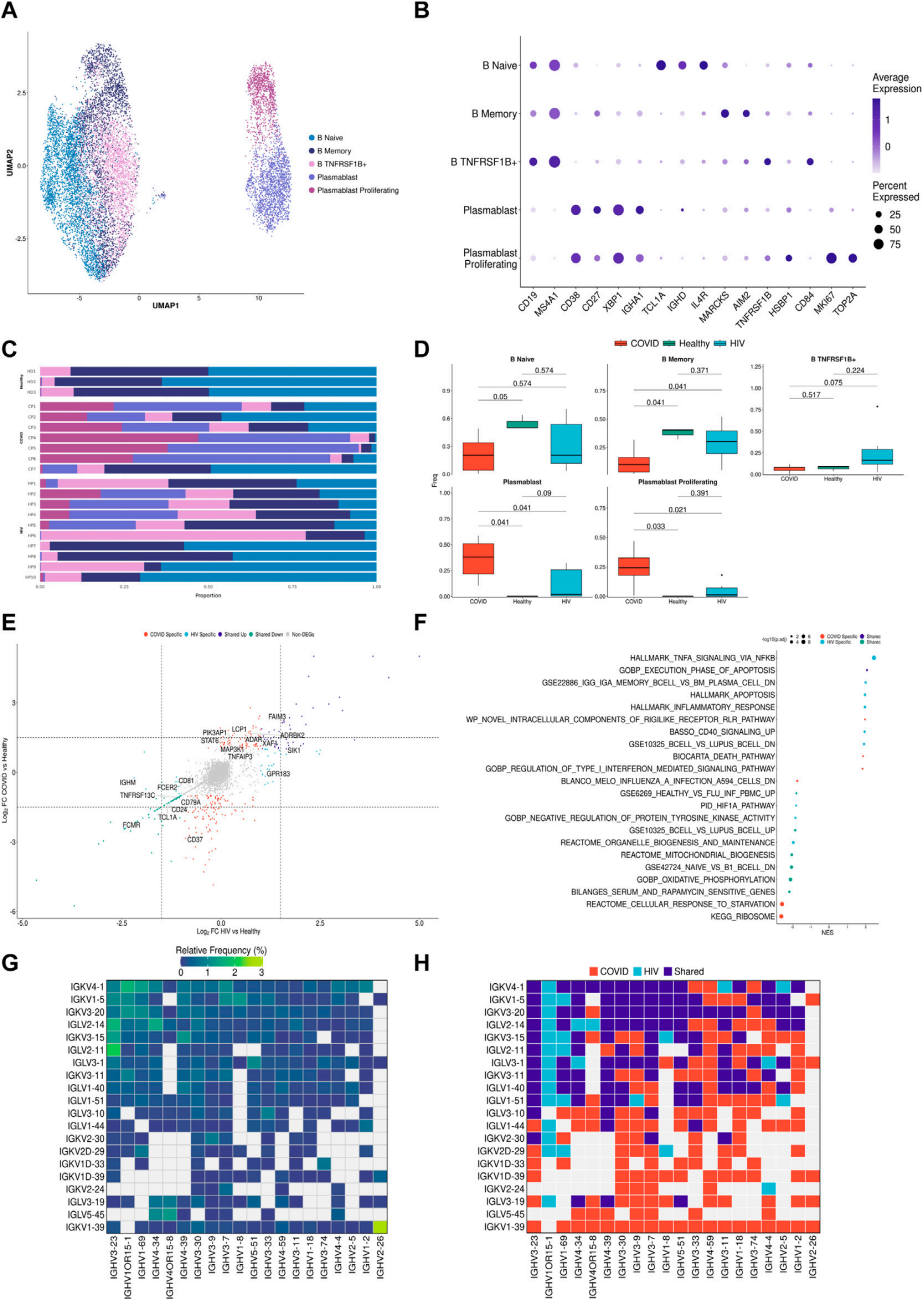
B cells in COVID-19 show more robust plasmablast response and antibody diversity relative to HIV-1. **(A)** UMAP embeddings of B cells colored by subtype. **(B)** Dot plot of canonical B cell marker expression across subtypes. **(C)** Stacked bar plots of the relative frequency of subtypes present in each patient. **(D)** Box plots of the proportions of B cell and plasmablast subsets across each disease condition. Proportions are computed for each patient by dividing their number of cells in each B cell/plasmablast subset by their total number of B cells + plasmablasts. *p*-values are computed with Wilcoxon signed-rank test with Holm-Bonferroni adjustment. **(E)** Double differential gene expression plot of genes that are differentially expressed between COVID-19 patients compared to healthy controls or differentially expressed between HIV-1^+^ patients compared to healthy controls. **(F)** Dot plot of enriched biological pathways from differentially expressed genes that were found to be upregulated (right, positive) or downregulated (left, negative) compared to healthy controls. **(G)** Heatmap of top 20 light chain (*Y*-axis) and heavy chain (*X*-axis) combinations found in HIV-1^+^ and COVID-19 patients. **(H)** Heatmap indicating the light chain/heavy chain combinations that are either unique to HIV-1^+^ (light blue), COVID-19 (red), or shared across the two diseases (dark blue).

**FIGURE 5:**
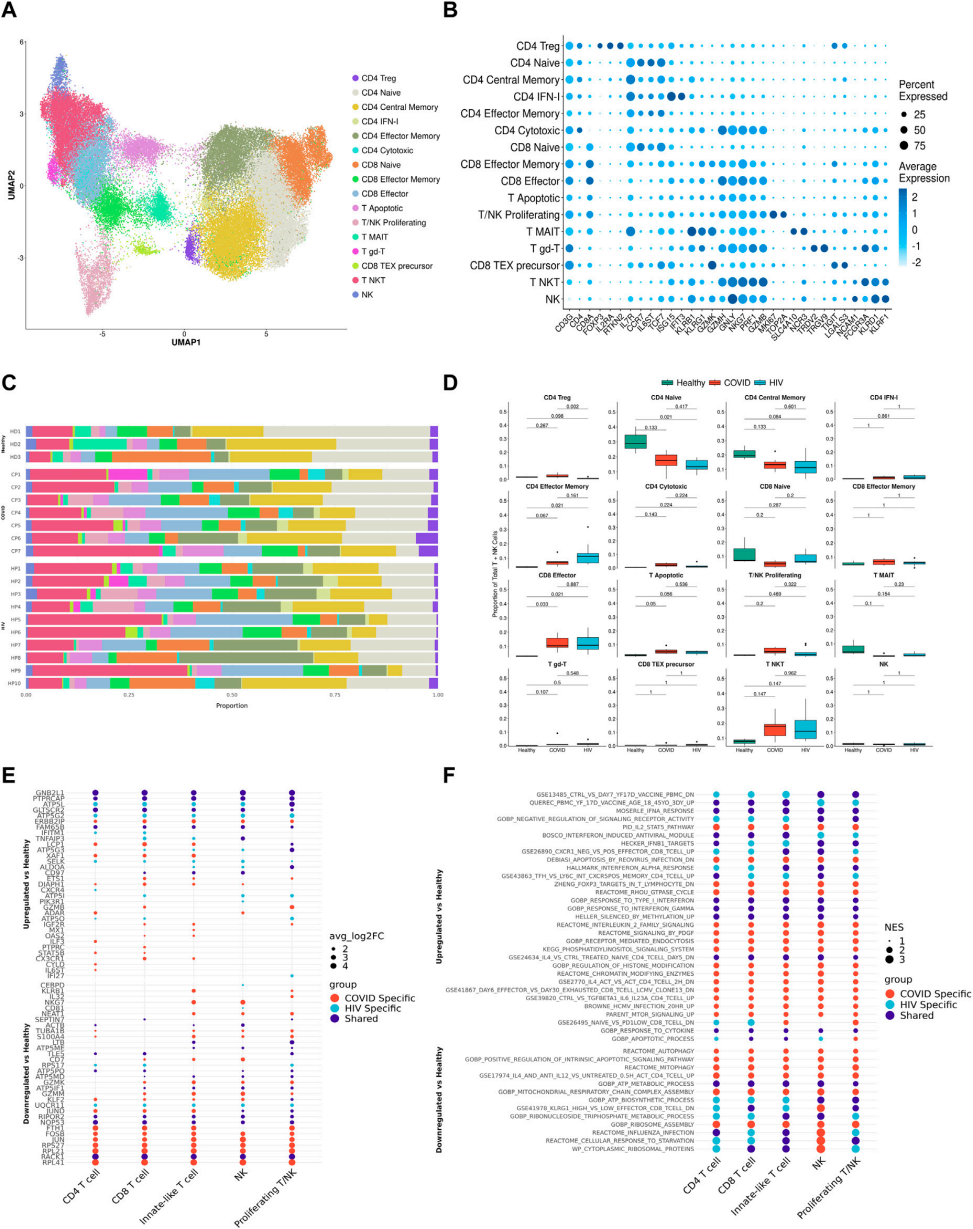
T cells in COVID-19 and HIV-1 show varied IFN-I and activation signatures. **(A)** UMAP embeddings of T cells colored by subtype. **(B)** Dot plot of canonical T cell marker expression across subtypes. **(C)** Stacked bar plots of the relative frequency of subtypes present in each patient. **(D)** Figure 5D: Box plots of the proportions of each T/NK cell subset across each disease condition. Proportions are computed for each patient by dividing their number of cells in each T/NK subset by their total number of T + NK cells. *p*-values are computed with Wilcoxon signed-rank test with Holm-Bonferroni adjustment. **(E)** Dot plots of the key genes differentially upregulated (top) or downregulated (bottom) compared to healthy controls. **(F)** Dot plot of enriched biological pathways from differentially expressed genes that were found to be upregulated (top) or downregulated (bottom) compared to healthy controls.

We computed frequencies of each cell type for each patient and performed Wilcoxon signed-rank tests (with Holm-Bonferroni adjustment for tests with multiple comparisons) to find significantly different compositions between pairs of patient types (HIV, COVID-19, and healthy). For broad cell types, frequencies are computed as a fraction of total PBMCs (i.e. Figure 2D). For lineage-specific cell types, frequencies are computed as a fraction of that specific lineage (e.g. Figures 3D, 4D, 5D).

### Cluster purity assessment

We utilized the ROGUE package (Liu et al., 2020) (v1.0) to assess purity of clusters determined by consensus labels. We calculated the expression entropy of each gene using the “SE_fun” method with “span” = 1.0. We calculated the ROGUE value of each consensus label across each patient using the “CalculateRogue” function with “platform” = “UMI”.

### Differential gene expression analysis and gene set enrichment analysis (GSEA)

To compare the relative similarities and differences of HIV-1 and COVID-19 gene expression, we performed differential gene expression analysis for either disease with respect to healthy controls. Differentially expressed genes were determined using a Wilcoxon Rank Sum test with Seurat’s “FindMarkers” function with the parameters “logfc.threshold” = 0 and “min.pct” = .1. *p* values were adjusted based on Bonferroni correction. We denoted differentially expressed genes (DEGs) with average log-2-fold change greater than 1 or less than −1 as differentially upregulated or downregulated, respectively. We performed GSEA on DEGs using the clusterProfiler package (v3.18.1) (Yu et al., 2012) with the “GSEA” function using default parameters using pathways from the MSigDB database (Subramanian et al., 2005).

### B cell chain analysis

We determined the frequency of heavy chain/light chain combinations using a method adopted from (Melms et al., 2021). We filtered B cells and plasmablasts to only the cells that expressed both heavy chain (IGVH) and light chain (IGVL) genes. These consisted of genes beginning with IGHG, IGHM, IGHA, or IGHE for heavy chain genes, and IGLV or IGKV for light chain genes. We counted the number of mRNA transcripts for each IGVH and each IGVL gene expressed on a per-cell basis, then assigned the most highly expressed IGVH and IGVL genes to be that cell’s IGVH-IGVL pairing. We categorized each combination as disease-specific if at least 1 cell expressed that combination in a given patient and shared if it was found in a patient from both diseases.

### Receptor-ligand analysis

To infer the putative receptor-ligand interactions between pairs of cell types, we utilized CellPhoneDB (Efremova et al., 2020). We first normalized raw count matrices to counts per 10,000 for each patient. We then performed CellphoneDB separately for each patient using the statistical method and default parameters, while supplying labels as the metadata for the 10 broad cell types. This was done to maintain biological accuracy, as feasible ligand-receptor interactions are only meaningful when measured within a given patient. We filtered out all ligand-receptor pairs with negative values, then merged interactions from patients of the same disease, treating each ligand-receptor/cell type combination as a unique interaction while preserving directionality (i.e. monocyte-NK is unique from NK-monocyte). This was done to capture the full spectrum of possible interactions across cell types. We averaged expression values and *p*-values for each interaction across patients. We repeated this process for all 25 consensus labels.

### IFN-I correlation analysis

We first compiled genes belonging to MSigDB pathways including the term “Type-I Interferon Signaling” into an IFN-I gene list. We identified DEGs between HIV-1^+^ and COVID-19 patients using the previously mentioned parameters and filtered them to keep only IFN-I related genes. We scored each gene module using the Seurat function “AddModuleScore”. To perform correlation analysis, we first used the SuperCell package (Okhotnikov et al., 2016) to group cells from each batch into supercells of 100 cells each using 5 K-Nearest-Neighbors and 2,000 variable genes and combined the resulting gene expression matrices from common diseases together. We then ran the “bicor” function from the WGCNA package (v1.70) (Langfelder and Horvath, 2008) on each gene belonging to the COVID-19 IFN-I module with each gene present in the COVID-19 supercell matrix, and extracted the top correlated genes (>.65). This was repeated for the HIV-1 IFN-I module and supercell matrix. We then performed GSEA enrichment on each set of top correlated genes.

## Results

### Consensus clustering approach corrects cell type labels and reveals additional cell subsets

A study comparing gene expression at the single-cell level following SARS-CoV-2 and HIV-1 infections has not been previously performed. Therefore, our analysis required that we integrate scRNA-seq data from different sources. Attempts to integrate scRNA-seq data from different sources (for example, to compare PBMCs) have mainly utilized manual supervision based on marker gene expression to assign cellular labels (Liu N. et al., 2021). These labels can be subjective and difficult to compare across different studies due to differences in label granularity and choice of markers (Liao et al., 2020; Wen et al., 2020). Here, we developed a reliable and accurate integration strategy to transfer labels from study one to another for cross-study analyses.

We aggregated publicly available scRNA-seq data from PBMCs derived from 7 severe COVID-19 (20, 829 cells) (Wilk et al., 2020), 10 HIV-1^+^ (70, 203 cells) (Kazer et al., 2020; Wang et al., 2020), and 3 healthy patients (20,534 cells) (10xGenomics, 2020) (Tables 1, 2, 3; Figure 1A, left). Our integration strategy is based on the combination of three different annotation approaches, namely manual annotation, correlation-based label transfer and deep-learning-based classification (Aran et al., 2019; Xu et al., 2021). We performed cell annotation using each of the three methods independently, and then integrated the three sets of labels to produce one final set of consensus labels (Figure 1A; Supplementary Figure S1A, S1B; see Materials and Methods). Our deep learning annotation resulted in high accuracy and purity across cell types (Figure 1B; Supplementary Figure S1C–S1E).

Our integration strategy resulted in 25 total cell types, consisting of 5 B cell subsets, 2 dendritic cell (DC) subsets, 2 monocyte subsets, 7 CD4^+^ T cell subsets, 8 CD8^+^ T cell subsets, and 1 natural killer (NK) cell subset (Figure 1C, bottom). For reference, 15 cell types had been identified in the COVID-19 scRNA-seq data source publication (the most detailed of the source publications) (Wilk et al., 2020), comprised of 4 B cell subsets, 2 DC subsets, 2 monocyte subsets, 2 CD4^+^ T cell subsets, 4 CD8^+^ T cell subsets, and 1 NK cell subset (Figure 1C, top). In the COVID-19 scRNA-seq data, we identified subsets amongst CD4^+^ T cells, CD8^+^ T cells, and unconventional T cells which were previously unclassified by the source publication: effector memory CD4^+^ T cells, cytotoxic CD4^+^ T cells, IFN-I^+^ CD4^+^ T cells (which express high levels of ISGs), regulatory CD4^+^ T cells (Tregs), naïve CD8^+^ T cells, precursor exhausted CD8^+^ T cells, natural killer T (NKT) cells, mucosal-associated invariant T (MAIT) cells, and apoptotic T cells. Using a confusion matrix, we identified subpopulations with disagreeing labels (between original and consensus) (Figure 1D). We then compared gene expression in these subpopulations, using the expression of canonical genes to confirm that our updated consensus labels were correct. This method corrected cells originally labeled as CD4^+^ T cells, B cells, and NK cells to their accurate labels: CD8^+^ T cells, plasmablasts, and unconventional T cells, respectively (Figure 1E; see Materials and Methods). Our labels also consistently displayed high cluster purity (>.75) according to their ROGUE score (Liu et al., 2020) (Supplementary Figure S1D). Overall, our consensus clustering approach allowed us to generate high-resolution labels with improved biological accuracy.

### Integrated immune landscape of PBMCs from COVID-19, HIV-1^+^ and healthy patients

We integrated all the single-cell data (7 COVID-19, 9 HIV-1^+^, and 3 healthy controls) into a single UMAP, where the 25 consensus clusters grouped into 10 major cell types (Figure 2A). The balanced distribution of cells across the three conditions demonstrated the successful integration of the data (Figure 2B, top). Of the four major cell clusters present in the UMAP, the top left cluster comprises CD4^+^ and CD8^+^ T cells, innate-like T cells (lymphocytes that express both the T-cell marker *CD3G* and NK-cell markers *GNLY* and *NKG7*), NK cells, and proliferating cells; the central and bottom clusters comprise primarily plasmablasts and B cells; and the rightmost cluster consists of monocytes and dendritic cells (DCs) (Figure 2B, bottom). Given that scRNA-seq results can vary due to confounding factors across batches, we explicitly regressed out any patient-specific effects to provide a more accurate representation of the distribution of cell types across patients (Figure 2C).

COVID-19 and HIV-1^+^ patients had elevated frequencies of CD8^+^ T cells compared to healthy controls (Figure 2D; Supplementary Figure S2), indicating the recruitment of inflammatory cells with both viral infections. However, DEG analysis, comparing healthy controls to patients with each disease, revealed that HIV-1^+^ patients uniquely exhibited substantial upregulation of *IL8*, *CCL3*, and *NFKBIA*, which have been implicated in the antiviral response and inflammation (Figure 2E), while patients with COVID-19 showed upregulation of *OAS2*, *XAF1*, and *MX1*, which are part of the type-I interferon (IFN-I) signaling pathway (Figure 2E; Supplementary Figure S3A). OAS proteins function to degrade double-stranded RNA viral intermediates during coronavirus replication (Choi et al., 2015). A recent genome-wide association study (GWAS) reported a significant association between genetic variants in human OAS genes and COVID-19 severity (Pairo-Castineira et al., 2021). Other IFN-associated genes, such as *ISG15*, *IFI27,* and *IFITM3*, were commonly upregulated (Supplementary Figure S3B). Patients with COVID-19 showed downregulation of genes involved in the AP-1 transcription factor pathway (including *JUN*, *JUNB*, *JUND*, and *FOSB)* as well as HLA genes (including *HLA-E*, *HLA-DRB1*, *HLA-DRA*, and *HLA-DPB1*) (Supplementary Figure S3C). We found a shared downregulation of *LTB*, which encodes lymphotoxin-B, an inflammatory protein that plays a role in lymphoid tissue development (Lu and Browning, 2014) and *KLF2*, which regulates the differentiation and function of immune cells (Jha and Das, 2017) (Figure 2E).

Gene set enrichment analysis (GSEA) on each set of DEGs revealed high enrichment for interferon-alpha/beta (IFN-α/β) signaling and interferon-gamma (IFN-γ) signaling in both COVID-19 and HIV-1^+^ PBMCs (Figure 2F). We also identified disease-specific enrichment of other signaling pathways. Most notably, PBMCs from COVID-19 patients were enriched in JAK-STAT and IL-4 signaling, which has been known to drive inflammation following infection (Lu et al., 2011; de la Rica et al., 2020; Satarker et al., 2021). PBMCs from COVID-19 patients also showed upregulated IL-10 production, an anti-inflammatory cytokine that is correlated with disease severity (Islam et al., 2021). In contrast, cells from HIV-1^+^ patients were enriched in CD40 signaling and CD4^+^ T cell activation, as well as apoptosis. We also found a shared downregulation of ribosome and oxidative phosphorylation (OXPHOS) pathways, which indicates that both infections cause a shift in metabolic function, potentially due to viral hijacking of cellular metabolic machinery or host responses. In conclusion, while our analysis of the immune landscape in COVID-19 and HIV-1 reveals a common theme of inflammation, IFN-I signaling, and metabolic reprogramming, even at the broadest level we begin to see differential enrichment of specific genes and biological pathways.

### Innate-induced inflammation is driven by different genes and cell-cell interactions in COVID-19 and HIV-1

We surmised that cell-type-specific analyses, based on the four main clusters in the UMAP (Figure 2A), could shed further light on the potential virus-specific immune specific pathways emerging in our results. Since a strong inflammatory monocyte response is a hallmark of both diseases, we first sought to closely examine the transcriptomic differences between DCs and monocytes in COVID-19 and HIV-1^+^ patients (rightmost cluster in UMAP, Figure 2A). We subsetted out only the DCs and monocytes and performed integration (removing patient-specific features, etc., as above) followed by clustering analysis based on gene expression. We identified 4 total clusters (Figure 3A): conventional dendritic cells (cDCs, *CD1C* ^high^ *FCER1A* ^high^ *CLEC10A* ^high^) (Collin et al., 2013), plasmacytoid dendritic cells (pDCs, *CLEC4C* ^high^ *IL3RA* ^high^ *TCF4* ^high^) (Collin et al., 2013), classical monocytes (*CD14* ^high^ *FCGR3A* ^low^) (Kapellos et al., 2019), and non-classical monocytes (*CD14* ^low^ *FCGR3A* ^high^) (Kapellos et al., 2019) (Figure 3B). We found that HIV-1^+^ patients exhibited significantly higher frequencies of non-classical monocytes compared to COVID-19 patients and healthy donors (adjusted *p*-values = 0.01 and 0.036, respectively) (Figures 3C, D), which have been shown to massively expand in the peripheral blood in response to immune activation by HIV-1 infection (Campbell et al., 2014). We did not find any significant sex-driven differences in any subset (Supplementary Figure S4A). While we did not find a significant difference in non-classical monocyte frequencies between acute and chronically-infected HIV-1^+^ individuals (adjusted *p*-value = 0.055) (Supplementary Figure S4B), male HIV-1^+^ individuals were found to have a higher frequency compared to female HIV-1^+^ individuals (adjusted *p*-value = 0.0159) (Supplementary Figure S4C). DEG analysis revealed a common inflammatory phenotype across both HIV-1 and COVID-19 which included known IFN-I signaling genes such as *IFITM3* and *IFI27* (Figures 3E, F). However, we once again found that most contributing genes were virus specific. Monocytes from HIV-1^+^ patients express high levels of genes associated with proinflammatory cytokines including *CXCL3, CCL3*, and *IL1B*, all of which play a role in the acute viral response and immune cell recruitment (Figure 3E; Supplementary Figure S4D). Monocytes from patients with COVID-19 express high levels of genes associated with inflammation including *IL17RA,* JAK-STAT-associated gene *STAT6*, and inflammatory protein-encoding genes *TNFRSF1B* and *ANXA2* (Supplementary Figure S4D). Interestingly, while cDCs in HIV-1^+^ patients did show a slight upregulation of *SAMHD1*, which encodes an antiretroviral protein reported to be effective in inhibiting early HIV-1 infection, *SAMHD1* gene expression was much more upregulated in cDCs from patients with COVID-19.

We utilized external scRNA-seq PBMC data to validate our findings, hereinafter referred to as the “validation dataset”. The validation dataset consisted of a COVID-19 dataset (Liu C. et al., 2021) of patients with severe COVID-19 (*n* = 5), critical COVID-19 (*n* = 25), and healthy donors (*n* = 16); in addition to a HIV dataset (Farhadian et al., 2022) of HIV^+^ individuals (*n* = 6) and healthy donors (*n* = 4). We first performed DEG analysis between COVID-19 and HIV-1 monocytes, assigning DEGs into two separate disease-specific monocyte signatures. We then performed GSEA on DEGs found between COVID-19 and HIV-1 monocytes in the validation dataset using the monocyte signatures, which revealed a significant enrichment (adjusted *p*-values = 2.87e-12 and 0.0480) (Supplementary Figures S4E, F), illustrating that DEGs found in our monocyte analysis were also enriched in the validation dataset.

GSEA analysis of monocyte DEGs revealed shared upregulation of inflammatory pathways such as IFN-I response and IFN-α/β signaling (Figure 3F). However, we also found a greater diversity in the inflammatory response associated with COVID-19 compared to HIV-1. Enrichment of IL-20, IL-2, IL-6, KIT, and JAK-STAT signaling pathways was unique to monocytes and DCs from patients with COVID-19, suggesting that innate immune cells may be much more active and cytotoxic in COVID-19 compared to in HIV-1 (Figure 3F). Several of these cytokines, including IL-6, have been found to be overexpressed and positively correlated with disease severity in COVID-19 patients (Costela-Ruiz et al., 2020; Weisberg et al., 2020; Jones and Hunter, 2021; Ma et al., 2021; Rubin et al., 2021).

Given the different functional profiles of DCs and monocytes between the two viral infections, and the frequent interactions of DCs and monocytes with the adaptive immune system as antigen presenting cells (APCs), we surmised that these cells may also play a divergent role in mediating the adaptive immune response. We applied CellphoneDB (Efremova et al., 2020) to determine putative receptor-ligand interactions based on gene co-expression patterns on pairs of cell types (Figure 3G). We found that DC-T cell and monocyte-T cell interactions were noticeably enriched in COVID-19 (Figure 3G). Further, cDCs from patients with COVID-19 had more frequent interactions with monocytes, NK cells, and T cells, while cDCs from HIV-1^+^ patients had more frequent interactions with plasmablasts. We next took a closer look at the CD4^+^ and CD8^+^ T cell receptor-ligand interactions with monocytes and DCs across the two infections (Figures 3H, I). We found a large number of costimulatory and inflammatory interactions shared across cell types and diseases, notably *CD28*−*CD86* (which provides a critical costimulatory signal for T cells) (Hui et al., 2017), *CD6*-*ALCAM* (which drives immune synapse formation and activation, and migration in CD4^+^ T cells) (Ampudia et al., 2020), and *TNFRSF1B*-*GRN* (which drives apoptosis and inflammation) (Ward-Kavanagh et al., 2016). We also found common enrichment of the inhibitory interaction *CD99*-*PILRA* (which curbs NK-like cytotoxicity). Migratory inhibitory factor (MIF), which promotes inflammation by elevating cell recruitment (Grieb et al., 2010), is known to be present in high concentrations in the peripheral blood of HIV-1^+^ patients (Regis et al., 2010). We found that the *MIF*-*CD74* interaction between cDCs/monocytes and CD4^+^/CD8^+^ T cells was unique and highly prevalent across HIV-1^+^ patients (Figures 3H, I). We also found the IFNγ- IFNγ receptor interaction between cDCs/monocytes and CD8^+^ T cells to be uniquely upregulated in HIV-1^+^ patients. On the other hand, the inflammatory *NOTCH2*-*IL24* interaction which induces *STAT1* and *STAT3* to regulate cell proliferation and survival (Ouyang and O'Garra, 2019) and the inhibitory interactions *CTLA4*-*CD86* and *HAVCR2*-*LGALS9* were unique to patients with COVID-19. Altogether, this analysis indicates that although innate-induced inflammation is present in both diseases, it is likely driven by very different genes and cell-cell interactions.

### COVID-19 exhibits a stronger plasmablast and antibody response compared to HIV-1

B cells are the primary effectors of the humoral antiviral immune response (Upasani et al., 2021). To investigate if B cells from COVID-19 and HIV-1^+^ patients exhibited distinct transcriptional signatures, we performed integration and clustering on B cell and plasmablast populations (central and bottom clusters in UMAP, Figure 2A), identified by upregulation of *CD19*/*MS4A1* and *CD38* respectively (Figure 4A). We found 5 total subpopulations: naïve B cells (*TCL1A* ^high^ *IGHD* ^high^ *CD27* ^low^), memory B cells (*TCLA1* ^low^ *CD27* ^high^ *AIM2* ^high^), *TNFRSF1B* ^+^ B cells (*TNFRSF1B* ^high^ *CD84* ^high^), plasmablasts (*CD38* ^high^ *XBP*1^high^), and proliferating plasmablasts (*CD38* ^high^ *MKI67* ^high^ *TOP2A* ^high^) (Figure 4B) (Sanz et al., 2019). Consistent with prior COVID-19 studies that show extensive plasmablast expansion in infected patients (Bernardes et al., 2020; De Biasi et al., 2020; Kuri-Cervantes et al., 2020), we found that patients with COVID-19 have significantly higher proportions of plasmablasts and proliferating plasmablasts (adjusted *p*-values = 0.041 and 0.033, respectively), and significantly lower proportions of naïve and memory B cells (adjusted *p*-values = 0.05 and 0.041, respectively), compared to healthy controls (Figures 4C, D). This was also corroborated in the validation dataset, as critical COVID-19 patients had a significant (adjusted *p*-value = 0.007) increase in plasmablasts compared to healthy donors (Supplementary Figure S5A). We did not find any sex-driven differences in any subset (Supplementary Figure S5B). HIV-1^+^ patients also had increased proportions of plasmablast and proliferating plasmablast subsets compared to healthy controls, but these responses were more moderate. While we did find a significant (adjusted *p*-value = 0.042) increase in plasmablast frequency in acutely-infected HIV-1^+^ individuals compared to healthy donors (Supplementary Figure S5C), we found a non-significant (adjusted *p*-value = 0.114) difference compared to chronically-infected HIV-1^+^ individuals, which may suggest that plasmablasts expand in response to acute HIV-1 infection and persist throughout chronic infection. In HIV-1^+^ patients, we found an enrichment of *TNFRSF1B* ^+^ B cells (Figure 4D), a subset of effector memory-like B cells with intermediate expression levels of memory B-cell marker genes (intermediate expression of *AIM2* and *CD27*) and upregulation of *TNFRSF1B* and *CD84*. *CD84* is associated with B cell proliferation, activation and signal transduction (Tangye et al., 2002), while *TNFRSF1B* encodes for a TNF-receptor protein known to induce TNF-mediated apoptosis. Plasmablasts from patients with COVID-19 expressed higher levels of *XBP1* and *SLAMF7* than plasmablasts from HIV-1^+^ patients, suggesting greater maturation (Supplementary Figure S5D).

Differential gene expression analysis in B cells and plasmablasts between patients with COVID-19 and HIV-1, relative to healthy controls (Figure 4E) revealed a common upregulation of *SIK1*, a gene that regulates cell cycling and plays a role in plasmablast maturation. We also found shared upregulation of genes involved in apoptosis and B-cell activation including *TNFAIP3*, *XAF1*, and *LCP1*, as well as *ADAR*, which has been implicated in viral RNA replication (Zhu et al., 2020). Interestingly, we found shared downregulation of several conventional B-cell markers including *CD24*, *CD37*, *CD40*, and *CD79a*, which play key roles in BCR signaling and B-cell regulation. We also found downregulation of *FCER2*, *FCMR*, *LTB*, and *TNFRSF13*, which help regulate cell differentiation and maintain cellular homeostasis. Taken together, these results suggest that B cells in both COVID and HIV-1 are actively responding to viral infection, and as a result they exhibit a drastic shift away from homeostasis.

We also observed COVID-19-specific enrichment of signaling genes such as *MAP3K1* (Figure 4E), which helps activate JNK and ERK pathways, and *STAT6*, which is involved in IL-4 and IL-13 signaling (de la Rica et al., 2020; Goel et al., 2021). We found upregulation of activation markers *CD80* and *CD40* in HIV-1 B cells (Supplementary Figure S5E). Apoptosis-associated pathways were enriched in both COVID-19 and HIV-1 B cells and plasmablasts (Figure 4F). Consistent with our cell proportion analysis (Figure 4C), we found that terms related to plasmablasts were positively enriched in both COVID-19 and HIV-1, while terms related to B cells were negatively enriched (Figure 4F). We also found several important pathways specific to HIV-1: CD40 signaling, which regulates the activation of the non-canonical NF-κB and JNK signaling pathways (Hömig-Hölzel et al., 2008); and TNF-α signaling *via* NF-κB, suggesting that NF-κB may play a central role in regulating the humoral immune response in HIV-1.

Seeking to explore the antibody diversity across the two viral infections, we mapped the top immunoglobulin light chain (IGVL) and immunoglobulin heavy chain (IGVH) combinations found in either COVID-19 or HIV-1 B cells and plasmablasts to determine the most frequent IGVL-IGVH pairings (Figure 4G). Of the top combinations, 150 were unique to COVID-19, 29 were unique to HIV-1, and 110 combinations were shared, suggesting that the humoral response to produce antibodies is not only stronger in COVID-19, but also more diverse (Figure 4H). Out of the top 20 IGVH and IGVL combinations, we found IGKV1-39/IGHV2-26 (a COVID-19 specific combination) to be the most frequent combination, which could offer insight into a potential broadly neutralizing antibody (bnAb) design.

### T cells in patients with COVID-19 and HIV-1 exhibit different IFN-I profiles

Integration and clustering of T-cell and NK cell populations (top left cluster in UMAP, Figure 2A) uncovered 16 subpopulations in total (Figures 5A, B). Across the 19 donors, two clusters of effector CD4^+^ T cells were present in higher proportions in both COVID-19 and HIV-1^+^ patients relative to healthy controls, but especially frequent in COVID-19 patients: IFN-I^+^ CD4^+^ T cells, expressing high levels of IFN-I-stimulated genes *ISG15* and *IFIT3*, as well as *IL7R*, all of which are reported to be upregulated by IFNβ in CD4^+^ T cells (Hoe et al., 2010); and cytotoxic CD4^+^ T cells, expressing cytotoxic genes *GZMH*, *GNLY*, *NKG7*, *PRF1*, and *GZMB* (Figures 5C, D). Effector CD8^+^ T cells were also enriched in all patient samples compared to healthy controls (Figures 5C, D). In contrast, we found a shared decrease in naïve and central memory CD4^+^ T cells and naïve CD8^+^ T cells in both COVID-19 and HIV-1^+^ patients (Figures 5C, D), suggesting that viral infection is polarizing both CD4^+^ and CD8^+^ T cells toward cytotoxicity programs, especially following SARS-CoV-2 infection. The NK population found in our data is CD56^dim^CD16^+^ (Figure 5B). We did not find a CD56^bright^CD16^-^ population, which is corroborated in the data source publications.

Gene enrichment analysis further revealed an upregulation of genes associated with T cell activation and inflammation in COVID-19 and HIV-1. T-cell activation genes *PTPRCAP* and *CD97* were consistently upregulated in T cells from both patients with COVID-19 and HIV-1^+^ patients. COVID-19 subsets additionally expressed *LCP1*, *STAT5B*, and *ILF3* (Figure 5E), which are involved in T-cell activation and signaling. Although T cells from both diseases express high levels of genes encoding for inflammatory proteins and chemokines, COVID-19 subsets expressed high levels of *GZMB* and *CXC3CR1* (Figure 5E), suggesting increased cytotoxicity and terminal effector function, while HIV-1 subsets showed upregulation of *CXCR4* and *TNFAIP3*, which modulate cell proliferation and initiate inflammatory immune responses, respectively. We also identified IFN-stimulated, disease-specific genes; for example, *XAF1* is specifically upregulated in COVID-19 patients while *IFITM1* is specifically upregulated in HIV-1^+^ patients. In addition to its antiviral capability, *XAF1* can enhance IFN-induced apoptosis. To validate these DEGs, we first performed DEG analysis on COVID-19 and HIV T cells, assigning DEGs into two separate disease-specific T-cell signatures. We then performed GSEA on DEGs found between COVID-19 and HIV-1 T cells in the validation dataset using the T-cell signatures, which revealed a significant enrichment (adjusted *p*-values = 3.01e-6 and 0.0110) (Supplementary Figures S6A, S6B), illustrating that DEGs found in our T-cell analysis were also enriched in the validation dataset. Enhanced activation and cytokine signaling, namely IFN-α and IFN-γ, were common to both COVID-19 and HIV-1 subsets. We found diverse signaling pathways and activation pathways associated with COVID-19 subsets, which were enriched in IL2/STAT5, PDGF, and mTOR signaling (Figure 5F). HIV-1 subsets, in contrast, exhibited a less terminally differentiated phenotype, and upregulated IFN-β signaling (Figure 5F).

Altogether, our results revealed a shared activated profile characterized by a robust IFN-I response in T cells from both COVID-19 and HIV-1^+^ patients, but we once again found distinct genes that regulate either response. These findings motivated further investigation into IFN-I signaling as detailed in following sections.

### IFN-I response is correlated with distinct signaling in COVID-19 versus HIV-1

**FIGURE 6:**
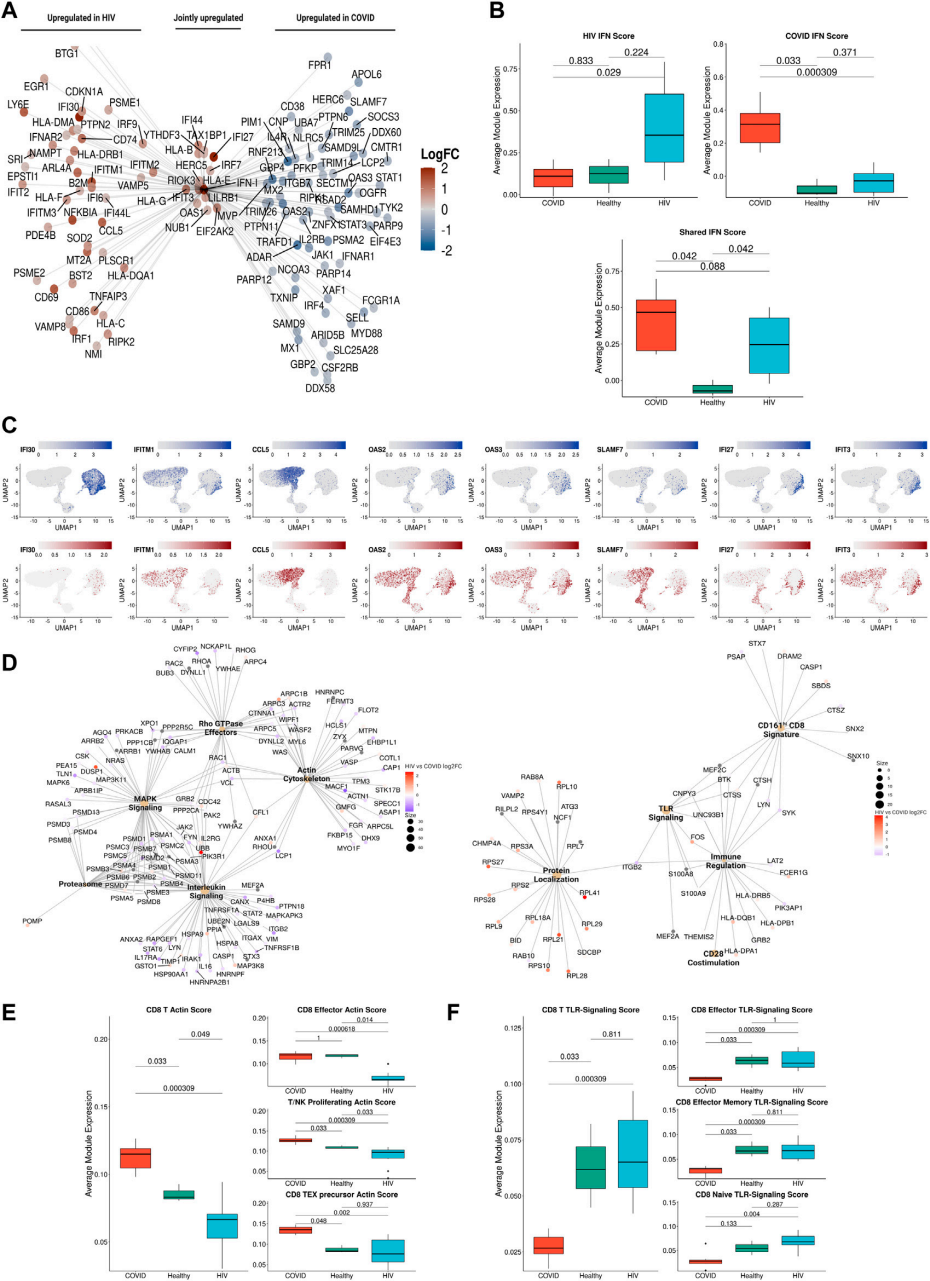
IFN-I signaling is correlated with divergent biological functions in COVID-19 versus HIV-1. **(A)** Network plot of genes related to IFN-I signaling that are differentially upregulated in COVID-19 (Right), HIV-1 (Left), or jointly upregulated compared to healthy controls. Genes are colored based on Log-2-fold change in expression in HIV-1^+^ versus COVID-19 patients. **(B)** Box plots of the average expression of HIV-1, COVID, and shared IFN-I module scores for each condition. *p*-values are computed with Wilcoxon signed-rank test with Holm-Bonferroni adjustment. **(C)** Normalized gene expression plots of IFN-I genes in COVID-19 (top) and HIV-1 (bottom). **(D)** Network plot of key pathways correlated with IFN-I signaling in COVID-19 (left) and HIV-1^+^ patients (right). Size of each center corresponds to the number of genes present in the pathway. Genes are colored based on Log-2-fold change in expression in HIV-1^+^ versus COVID-19 patients. **(E)** Box plots of the average expression of CD8^+^ T cell actin polymerization module scores for each disease condition (left). Box plots of the average expression of CD8^+^ effector, T/NK proliferating, and CD8^+^ Tex precursor scores for each disease condition (right). *p*-values are computed with Wilcoxon signed-rank test with Holm-Bonferroni adjustment. **(F)** Box plots of the average expression of CD8^+^ T cell TLR-signaling module scores for each disease condition (left). Box plots of the average expression of CD8^+^ effector, CD8^+^ effector memory and CD8^+^ naive TLR-signaling scores for each disease condition (right). *p*-values are computed with Wilcoxon signed-rank test with Holm-Bonferroni adjustment.

IFN-I signaling plays pleiotropic roles during viral infection, including stimulation of T-cell survival, proliferation, and memory formation. Given the consistent observation of IFN-I signatures across all cell subsets in both COVID-19 and HIV-1, we reasoned that IFN-I may regulate the distinct immune responses against the two diseases. Closer examination of specific IFN-I-associated genes revealed stark disease-specific enrichment, as nearly all IFN-I-associated genes were differentially upregulated in COVID-19 or HIV-1 (Figure 6A). We categorized all IFN-I-associated genes into three IFN-I modules: HIV-1-specific, COVID-19 specific, or shared (Figure 6B). In HIV-1, monocytes and cDCs exhibited the highest associated IFN-I score, suggesting that they may be primarily responsible in driving the IFN-I response (Supplementary Figure S7A). We examined the cell type-specific expression of IFN-I associated genes and found that the effector molecule *CCL5* was jointly expressed in CD8^+^ T cells in both diseases. In addition, IFI30 was specifically upregulated in monocytes during HIV-1 infection, whereas *SLAMF7* and *IFIT3* were upregulated in plasmablasts and T cells from COVID-19 patients respectively (Figure 6C). In the validation dataset, we found many HIV-1-specific IFN-I genes (such as *IFI30*, *IRF9, ISG15*, and *IFITM2*) to be upregulated by HIV-1^+^ individuals (Supplementary Figure S7B), as well as many COVID-19-specific IFN-I genes (such as *IRF4*, *MX1*, *IFNAR1*, and *OAS3*) to be upregulated by COVID-19 patients (Supplementary Figure S47C)

Using bicorrelation analysis on disease-specific IFN-I genes, we found a much higher number of IFN-I-correlated genes and enriched pathways in patients with COVID-19 compared to HIV-1 (main pathways shown in Figure 6D). Notably, COVID-19 IFN-I-correlated genes showed enrichment for MAPK signaling and interleukin signaling, which have been implicated in inflammation, thrombosis, and pulmonary injury and cytokine storms, respectively (Figure 6D, left). Proteasomal genes were also enriched among the IFN-I-correlated genes in COVID-19 patients; the ubiquitin-proteasome system (UPS) facilitates the production of viral proteins (including in SARS-CoV), and has been proposed as a target for COVID-19 treatment (Longhitano et al., 2020).

We also found an enrichment of the Rho GTPase metabolic pathway in COVID-19 patients (Figure 6D, left). GTPase activation contributes to immune cell activation and migration, as well as coagulation, often resulting in severe lung injury (Abedi et al., 2020a). Rho GTPases regulate a diversity of cellular processes, including cell migration and cell cycling, as well as modulation of cytoskeletal rearrangements (Hodge and Ridley, 2016). The overlap between Rho GTPase and actin cytoskeleton genes in Figure 6D confirms this relationship. The actin cytoskeleton signature was significantly (adjusted *p*-values = 0.033 and 0.000309) upregulated in COVID-19 patients compared to HIV-1^+^ individuals and healthy donors (Figure 6E, left), and was especially pronounced in effector CD8 T cells, proliferating T and NK cells, and precursor exhausted CD8 T cells (Figure 6E, right and Supplementary Figure S8A). In contrast, pathways correlated with IFN-I signaling in HIV-1^+^ patients were all related to immune activation, including TLR signaling and CD28 co-stimulation (Figure 6D). Persistent viral antigen presentation in HIV-1^+^ patients can induce chronic inflammation and constitutive TLR signaling, partly *via* LPS translocation which can lead to disease progression (Brenchley et al., 2006; Meier and Altfeld, 2007). The TLR signaling signature was significantly (adjusted *p*-value 0.000309) upregulated in HIV + individuals compared to COVID-19 patients (Figure 6F, left), and was especially pronounced in effector, effector memory, and naïve CD8 T cells (Figure 6F, right and Supplementary Figure S8B). Overall, our analysis demonstrates the differential IFN-I signaling following SARS-CoV-2 and HIV-1 infections and suggests that IFN-I signaling activates a greater diversity of immune cell functions in COVID-19 compared to HIV-1 (Lee and Shin, 2020; Schreiber, 2020).

### Metabolic differences between T cells in COVID-19 and HIV-1

**FIGURE 7:**
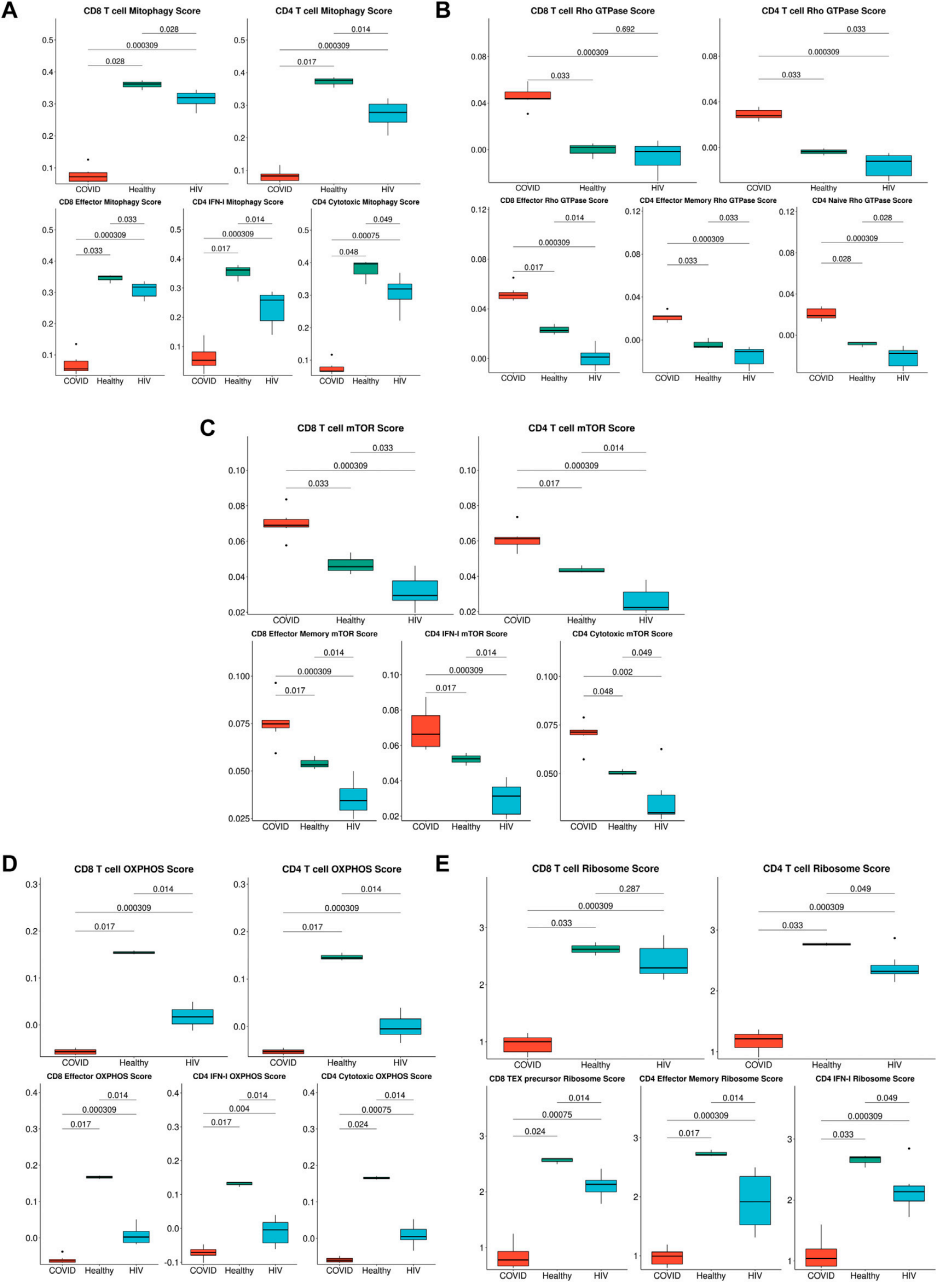
Metabolic differences associated with HIV-1 and COVID-19. **(A)** Box plots of the average expression of the mitophagy signature score in CD8^+^ and CD4^+^ T cells across each disease condition (top), Box plots of the average expression of CD8^+^ effector, CD4^+^ IFN-I and CD4^+^ cytotoxic mitophagy signature score for each disease condition (bottom). *p*-values are computed with Wilcoxon signed-rank test with Holm-Bonferroni adjustment. **(B)** Box plots of the average expression of the Rho GTPase signature score in CD8^+^ and CD4^+^ T cells across each disease condition (top), Box plots of the average expression of CD8^+^ effector, CD4^+^ effector memory, and CD4^+^ naïve Rho GTPase signature score for each disease condition (bottom). *p*-values are computed with Wilcoxon signed-rank test with Holm-Bonferroni adjustment. **(C)** Box plots of the average expression of the mTOR score in CD8^+^ and CD4^+^ T cells across each disease condition (top), Box plots of the average expression of CD8^+^ effector memory, CD4^+^ IFN-I, and CD4^+^ cytotoxic mTOR signature score for each disease condition (bottom). *p*-values are computed with Wilcoxon signed-rank test with Holm-Bonferroni adjustment. **(D)** Box plots of the average expression of the OXPHOS signature score in CD8^+^ and CD4^+^ T cells across each disease condition (top), Box plots of the average expression of CD8^+^ effector, CD4^+^ IFN-I, and CD4^+^ cytotoxic OXPHOS signature score for each disease condition (bottom). *p*-values are computed with Wilcoxon signed-rank test with Holm-Bonferroni adjustment. **(E)** Box plots of the average expression of the ribosome signature score in CD8^+^ and CD4^+^ T cells across each disease condition (top), Box plots of the average expression of CD8^+^ Tex precursor, CD4^+^ effector memory, and CD4^+^ IFN-I ribosome signature score for each disease condition (bottom). *p*-values are computed with Wilcoxon signed-rank test with Holm-Bonferroni adjustment.

The strong correlation of COVID-19 IFN-I signaling with Rho GTPase signaling suggested that enhanced IFN-I signaling in T cells could give rise to divergent metabolic profiles. Supporting this, two recent studies demonstrated that cellular metabolism is intimately linked to Rho GTPase activation and actin cytoskeleton organizations (Hu et al., 2016; Wu et al., 2021). Our analysis also consistently revealed pathways associated with apoptosis and impaired metabolic function (Figure 5F). We hypothesized that SARS-CoV-2 and HIV-1 infections may also induce distinct metabolic signatures in immune cells. We built gene modules from the differentially enriched metabolism-associated pathways (Figure 5F) and scored their expression in T cells. In addition to uncovering consistent metabolic shifts in T cells overall, we also found significant shifts in cytotoxic T cell subsets. We found that T cells in COVID-19 patients exhibited significantly lower mitophagy (Figure 7A, top), a process that maintains cellular homeostasis by removing damaged or dysfunctional mitochondria (Chen et al., 2020a) compared to immune cells in HIV-1^+^ patients and healthy controls. This difference was especially pronounced in more cytotoxic subsets (Figure 7A, bottom). We confirmed the high expression of Rho GTPase activity in COVID-19 T cells (Figure 7B, top), a trend that was consistent with both effector CD8^+^ T cells and effector memory CD4^+^ T cells (Figure 7B, bottom). We found that T cells in patients with COVID-19 showed increased mTOR pathway activity, while T cells in HIV-1^+^ patients showed downregulated mTOR activity, compared to healthy controls, (Figure 7C, top). The mTOR pathway regulates cell proliferation and survival, as well as CD4^+^ T cell and B cell responses (Ye et al., 2017; Akbay et al., 2020). HIV-1 infection has been shown to interfere with mTOR signaling, which usually results in diminished mTOR expression levels in immune cells, particularly in CD4^+^ T cells (Akbay et al., 2020). Cytotoxic CD4^+^ T cells from HIV-1^+^ patients had significantly downregulated mTOR expression types (Figure 7C, bottom). These results indicate that distinct IFN-I-signaling pathways give rise to different virus-specific metabolic signatures in immune cells. In the validation dataset, we found consistent metabolic trends in T cells; namely, we found that COVID-19 T cells downregulate key mitophagy genes *FUNDC1*, *PINK1*, and *CSNK2B* (Supplementary Figure S9A) and upregulate key Rho GTPase genes *RHOA*, *RHOBTB1*, and *ARAP3* (Supplementary Figure S9B) as well as *MTOR* (Supplementary Figure S9C) compared to HIV-1^+^ individuals. Finally, we found shared HIV-1 and COVID-19-associated downregulation of genes associated with ATP biosynthesis, OXPHOS, and ribosome assembly, indicating a common major metabolic shift in T cells from both diseases (Figure 7D, E).

## Discussion

**FIGURE 8:**
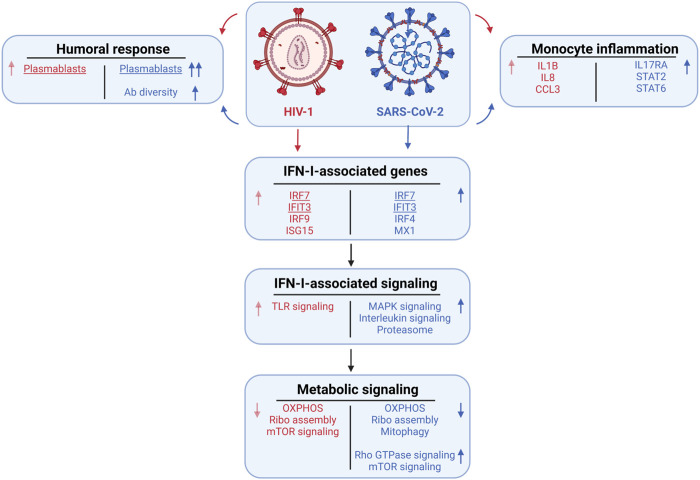
Summary of the major cellular shifts in COVID-19 compared to HIV-1.

Here, we sought to identify disease-specific viral pathways in two inflammatory diseases with different pathophysiologies. We designed a consensus annotation method to generate a high-quality unified cellular atlas of the immune landscape of PBMCs from COVID-19 and HIV-1^+^ patients. Our atlas highlights shared and contrasting signatures of humoral immune responses, inflammation, IFN-I signaling, and metabolism, demonstrating how these processes are conserved or divergent between viruses with distinct pathologies (summary in Figure 8). In addition to shedding light on the divergence of disease-specific viral clearance pathways, our strategy for integrating different sets of scRNA-seq data (from different tissues, organs, or diseases) will yield a valuable annotation resource for diseases less well studied at the single cell level.

We found a consistent inflammatory signature in innate immune cells, highlighted by IFN-I and cytokine-mediated signaling, across patients with COVID-19 and HIV-1 (Bieberich et al., 2021; Hasan et al., 2021; Liu N. et al., 2021). This is corroborated by conclusions from Kazer et al. and Wilk et al.; the former reported the enrichment of CXCL10^+^ inflammatory monocytes in HIV-1^+^ individuals, while the latter reported the enrichment of IFN-I-expressing inflammatory monocytes in COVID-19 patients. Further analysis of the types and frequencies of cellular communications among immune cells revealed the disease-specific inflammatory and cytotoxic molecules that drive the innate immune response in either disease. We hypothesize that excess inflammation induced by SARS-CoV-2 infection may be driving inhibitory and apoptotic programs. Namely, we found an enrichment of two COVID-19-unique inhibitory interactions between APCs and CD8^+^ T cells, *CTLA4/CD86* and *HAVCR2/LGALS9*, which may be out of necessity to curb the heightened inflammation present in severe COVID-19 (Figure 3I). We also found the specific downregulation of *NKG7* and *NEAT1* in innate-like T cells and NKs in COVID-19 patients. (Figure 5E). Since *NKG7* is important for cytotoxic degranulation and downstream inflammation and *NEAT1* is an activator of the NLRP3 inflammasome, their downregulation may indicate an induced shift away from an inflammatory state in COVID-19 patients (Chen et al., 2019; Malarkannan, 2020). The enrichment of apoptotic T cells in both diseases may be a consequence of heightened inflammation. Our findings are consistent with previous studies demonstrating that HIV-1 infection leads to apoptosis of uninfected bystander cells (Garg et al., 2012) as well as the observation that severe COVID-19 patients frequently experience lymphopenia (Chen et al., 2020b). Interestingly, we found a significant (adjusted *p*-value = 0.003) increase in Tregs in COVID-19 patients compared to HIV-1^+^ individuals. FOXP3^+^ Tregs have been reported to be expanded in severe COVID-19 infection (Chen X. et al., 2020) and may contribute to poor outcomes by suppressing antiviral T cell responses while also secreting proinflammatory cytokines (Galván-Peña et al., 2021). On the flip side, Tregs are susceptible to HIV infection, and decrease in quantity over the course of infection (Eggena et al., 2005; Chevalier and Weiss, 2013).

IFN-I plays a critical role in priming innate and adaptive immune responses during both SARS-CoV-2 and HIV-1 infection, as well as limiting viral replication and promoting effector cell function (Sugawara et al., 2019; Schreiber, 2020). IFN-I signaling has been characterized as generally beneficial in both acute SARS-COV-2 and HIV-I infection (Sandler et al., 2014; Abraham et al., 2016; Lavender et al., 2016; Utay and Douek, 2016; Wang et al., 2017; Lee et al., 2020; Galani et al., 2021). As a result, IFN-I treatment has been used to treat both infections, with moderate success (Lavender et al., 2016; Banday et al., 2022). However, during the late stages of chronic viral infection, IFN-I signaling shifts toward a pathogenic role by contributing to systemic inflammation (Teijaro et al., 2013; Wilson et al., 2013; Utay and Douek, 2016; Soper et al., 2017). The precise role of IFN-I at the single-cell level in COVID-19 (which results in an acutely controlled infection) and HIV-1 (which results in a chronic infection) is still unclear.

While IFN-I signaling was upregulated in both COVID-19 and HIV-1^+^ patients relative to healthy controls, our analysis suggests a more polyfunctional role for IFN-I in COVID-19. IFN-I signaling in COVID-19 was more intimately tied to important cellular functions such as cell signaling, motility, and cytokine secretion. In support of our findings, previous studies have found that exposure to IFN-I results in upregulation of MAPK signaling cascades (Zhao et al., 2011). While MAPK signaling regulates important functions such as cellular proliferation and survival, further studies are needed to investigate whether IFN-I-mediated MAPK signaling in COVID-19 contributes to the antiviral immune response or apoptosis (Zhang and Liu, 2002). Previous studies have reported an antagonistic relationship between IFN-I and IL-1, the prototypical proinflammatory cytokine (Guarda et al., 2011; Mayer-Barber and Yan, 2017). Interestingly, we found that IFN-I signaling in COVID-19 patients is highly correlated with immune-activating cytokine signaling pathways such as IL-2, IL-16, and IL-17, which could provide novel insights on the coregulatory relationship of IFN-I with other effector cytokines. In contrast, we found a much narrower scope of highly correlated genes and pathways in HIV-1^+^ patients, which could suggest an inflammation-specific role for IFN-I. Overall, our findings show that while the IFN-I response is robust in both diseases, the responses are tied to drastically different biological functions in HIV-1 compared to COVID-19, with the latter featuring a much more diverse spectrum of cellular responses. In agreement with our findings, recent analyses integrating a genome-wide association study (GWAS) and a transcriptome-wide association study (TWAS) suggested that the IFN response could determine COVID-19 severity (Pairo-Castineira et al., 2021). Our findings are also consistent with those from the data source publications: Kazer et al. found a core module of interferon-associated genes in HIV-1^+^ individuals that were highly expressed in multiple cell types; Wang et al. found a subset of CD8^+^ T cells expressing high levels of IFN-I associated-genes to be enriched in HIV-1^+^ individuals; Wilk et al. found a wide range of IFN-I-associated genes that were highly expressed by COVID-19 patients across nearly all cell types.

Notably, our analysis also revealed disease-specific altered metabolism profiles, specifically that enhanced IFN-I signaling in T cells gave rise to divergent metabolic profiles. We characterized a decrease in OXPHOS and ribosome biogenesis in response to both SARS-CoV-2 and HIV-1 infection. Virus-induced reduction of OXPHOS has been previously characterized in other diseases and could be a result of oxidative stress triggered by mitochondrial clustering (Khan et al., 2015). Viral hijacking of ribosomal function is also crucial to viral replication and survival in the host (Li, 2019). Prior studies have suggested that SARS-CoV-2 may hijack the host cell’s mitochondria, resulting in a reduction of ATP biosynthesis (Ganji and Reddy, 2021). Disruption of these viral interactions could be advantageous for COVID-19 and HIV-1 treatment.

Previous studies have reported that SARS-CoV-2 can activate the coagulation cascade in the blood, which could lead to a reduction in mitophagy (Ganji and Reddy, 2021). This altered rate of mitophagy forces cells to adopt apoptosis as an alternative, which could explain the elevated levels of apoptosis as well as apoptotic T cells seen in COVID-19. General and specific mitophagy were both reported to be important for T-cell homeostasis, function, and differentiation. Deficiency in this process can lead to cell death, which may also explain the cell apoptosis and lymphopenia experienced by severe COVID-19 patients (Pua et al., 2009; Kovacs et al., 2012; Watanabe et al., 2014; Botbol et al., 2016; Gassen et al., 2021).

Our observation of upregulated Rho GTPase signaling in COVID-19 patients is in line with the potential use of Rho kinase inhibitors to treat COVID-19; Rho kinase inhibitors can restore the activity and level of ACE2 which is inhibited by SARS-CoV-2 without increasing the risk of infection (Abedi et al., 2020b). A recent study demonstrated that small GTPase RhoA activation drives increased cellular glycolytic capacity (Wu et al., 2021) which is typically associated with reduced mitochondrial metabolism; this result is also in agreement with the upregulation of Rho GTPase and disrupted mitochondrial function in COVID-19 patients. Moreover, Rho GTPases have been linked to additional key metabolic controls such as mTOR signaling pathways, which are specifically upregulated in COVID-19 patients (Senoo et al., 2019; Mutvei et al., 2020). Targeting mTOR may help to regulate T cells by induction of autophagy without apoptosis, reduce viral replication, restore T-cell function, and decrease cytokine storms (Mashayekhi-Sardoo and Hosseinjani, 2021).

The identification of various molecular pathways known to regulate COVID-19 pathophysiology, many of which are under consideration for COVID-19 treatment, supports our analysis and results. For instance, we found enrichment of JAK-STAT signaling, IL-4 signaling, and IL-6 signaling in COVID-19 patients. Three JAK inhibitors that reduce excessive inflammation (Baricitinib, Tofacitinib, and Ruxolitinib) have been used to treat COVID-19 patients, among which Baricitinib and Tofacitinib are recommended for hospitalized patients who require high-flow oxygen or non-invasive ventilation according to NIH COVID-19 Treatment Guidelines (Satarker et al., 2021). In addition, Dupilumab, an IL-4Rα inhibitor, was also reported useful for treating COVID-19 patients (Thangaraju et al., 2020). Furthermore, IL-6R inhibitors Sarilumab and Tocilizumab were also shown to be beneficial for COVID-19 patients and were recommended for use in hospitalized patients who require supplemental oxygen, high-flow oxygen, non-invasive ventilation, or invasive mechanical ventilation by NIH COVID-19 Treatment Guidelines. Several COVID-19 patients in our data were treated with Azithromycin (Table 4), which induces an immunomodulatory effect intended to decrease pro-inflammatory cytokine production. Despite this, we found consistent inflammatory signatures in COVID-19 PBMCs (Figures 2E, F). We did not find a significant (*p*-values = 0.4, 0.857, and 1) difference between expression of stress-associated genes *FOSB, JUND*, or *NEAT1* as a result of treatment (Supplementary Figures S10A, S10B). Several HIV-1^+^ patients were treated with various antiretroviral therapies (ART) (Table 4). ART inhibits HIV-1 replication and slows the elimination of memory CD4^+^ T cells and memory B cells (Quiros-Roldan et al., 2012), which may explain the similar frequencies of memory CD4^+^ T cells and memory B cells in HIV-1^+^ individuals when compared to COVID-19 patients and healthy donors, respectively (Figures 3D, 4D).

**TABLE 4:** Treatment information for patients analyzed.

ID	Disease	Treatment
C1	Severe COVID-19	Azithromycin
C2	Severe COVID-19	None
C3	Severe COVID-19	Azithromycin
C4	Severe COVID-19	Azithromycin
C5	Severe COVID-19	None
C6	Severe COVID-19	None
C7	Severe COVID-19	None
P1	HIV	None
P2	HIV	None
P3	HIV	None
P4	HIV	None
Q1	HIV	GENVOYA
Q2	HIV	DESCOVY + TRUVADA + PREZISTA + PREZCOBIX + NORVIR
Q3	HIV	Triumeq
Q4	HIV	ODEFSEY + TIVICAY
Q5	HIV	None
Q7	HIV	JULUCA

In conclusion, our study provides a comprehensive comparison of the immunological landscape of SARS-CoV-2 and HIV-1 infections in humans. The high resolution of single-cell RNA sequencing, diversity of patient samples, and diverse datasets allowed us to dissect important shared and disease-specific features that may inform the next-generation of antiviral treatments. Through cell type-specific analysis, we found a common enrichment of activated B cells and plasmablasts, inflammatory monocyte and effector T cell subsets, and cytokine signaling that appear to drive the antiviral response to SARS-CoV-2 and HIV-1. We also found that DCs and monocytes were highly interactive with adaptive immune cells in both diseases, but that innate cells in COVID-19 appear to be more capable of immunosuppressive function through CTLA-4 and TIM-3-mediated interactions. We also report that the cytokine response was more diverse in COVID-19 patients, which is highlighted by IL-2, IL-4, and IL-20 signaling, while HIV-1^+^ individuals primarily exhibited high levels of NF-kB signaling.

### Limitations

While our analysis revealed new insights into both COVID-19 and HIV-1, our study is limited by the availability and clinical annotation of relevant datasets, particularly the scarcity of publicly available peripheral immune data of HIV-1 infection. As a result, the HIV-1^+^ individuals in this study have varied clinical and demographic backgrounds (Tables 2, 3). Despite these factors, the data analyzed from HIV-1^+^ individuals span both acute and chronic stages of infection and various levels of viral load (since data were collected from HIV-1^+^ individuals from Kazer et al. at multiple timepoints during both early and late infection), thus providing a holistic representation of the peripheral response to HIV-1 infection. To address the possible transcriptomic variances due to differences in patient background and sample collection batch, we applied rigorous and sensitive integration and explicitly supplied patient identity and sample batch as variables to regress out their effects (Korsunsky et al., 2019). While there are undoubtedly differences between stages of HIV infection, we consistently found more drastic changes in gene signatures and cellular interactions when specifically contrasting COVID-19 and HIV-1 compared to contrasting the state of HIV-1 infection (Supplementary Figs 10C, D), indicating that our results are in fact representative of the biological differences between the two diseases. We further validated our findings in external COVID-19 and HIV-1 scRNA-seq datasets. Finally, our study emphasizes the *in silico* reanalysis of previously published data using different methods to uncover novel disease biology. While we suggest that numerous cellular subsets, genes, and signaling pathways may be critical in regulating either or both diseases, further experiments to validate such findings are necessary.

## Data Availability

Publicly available datasets were analyzed in this study. This data can be found here: https://singlecell.broadinstitute.org/single_cell/study/SCP256 https://www.ncbi.nlm.nih.gov/geo/query /acc.cgi?acc=GSE157829 https://www.ncbi.nlm.nih.gov/geo/query/acc.cgi?acc=GSE150728 https://www.10xgenomics.com/resources/datasets? query=&page=1&configure%5Bfacets%5D%5B0%5D=chemistryVersionAndThroughput& configure%5Bfacets%5D%5B1%5D=pipeline.version&configure%5BhitsPerPage%5D=500& configure%5BmaxValuesPerFacet%5D=1000&menu%5Bproducts.name%5D= Scripts to generate the main figures are available at https://figshare.com/s/28d4bcdd822a3de29be8.

## Supplementary material

SUPPLEMENTARY FIGURE S1 Assessment of scANVI training and consensus clustering. **(A)** Hierarchical clustering of original labels derived from Ren Cell 2021. **(B)** Table depicting the merging of labels from Ren Cell 2021 that were used for scANVI training. **(C)** Balanced accuracies of scANVI-derived labels compared against their original label from Ren Cell 2021. **(D)** ROGUE scores of final consensus labels. Error bars denote variation across patients. **(E)** Confusion matrix comparing the observed labels provided by scANVI (*X* axis) and predicted labels (*Y* axis).

SUPPLEMENTARY FIGURE S2 Differential abundance of major PBMC subsets across sex. Box plots of the proportions of major PBMC subsets across sex. Proportions are computed for each patient by dividing their number of cells in each subset by their total number of PBMCs. P-values are computed with Wilcoxon signed-rank test.

SUPPLEMENTARY FIGURE S3 Gene expression heatmaps across COVID-19, HIV-1^+^, and healthy patients. **(A)** Heatmap of selected genes found to be upregulated in COVID-19 patients from Zhu Immunity 2020 (Zhu et al., 2020) plotted across cell types and patient conditions. **(B)** Heatmaps of selected genes found to be upregulated in COVID-19 patients from Xu Cell Discovery 2020 (Xu et al., 2020) plotted across cell types and patient conditions. **(C)** Heatmaps of selected genes found to be downregulated in COVID-19 patients from Xu Cell Discovery 2020 (Xu et al., 2020) plotted across cell types and patient conditions.

SUPPLEMENTARY FIGURE S4 Differential abundance, gene expression, and signature enrichment of monocytes. **(A)** Box plots of the proportions of myeloid cell subsets across sex. Proportions are computed for each patient by dividing their number of cells in each DC/monocyte subset by their total number of DCs + monocytes. *p*-values are computed with Wilcoxon signed-rank test. **(B)** Box plots of the proportions of myeloid cell subsets across acutely and chronically infected HIV-1^+^ individuals. Proportions are computed for each patient by dividing their number of cells in each DC/monocyte subset by their total number of DCs + monocytes. *p*-values are computed with Wilcoxon signed-rank test with Holm-Bonferroni adjustment. **(C)** Box plots of the proportion of myeloid cell subsets across sex for HIV-1^+^ individuals. Proportions are computed for each patient by dividing their number of cells in each DC/monocyte subset by their total number of DCs + monocytes. P-values are computed with Wilcoxon signed-rank test. **(D)** Box plots of average gene expression for key monocyte genes, plotted for monocytes. *p*-values are computed with Wilcoxon signed-rank test. **(E)** GSEA enrichment plot of DEGs found to be upregulated by HIV-1 monocytes compared to COVID-19 monocytes from the validation dataset, scored on DEGs found to be upregulated by HIV-1 monocytes compared to COVID-19 monocytes from analysis. *p*-values are adjusted with Benjamini-Hochberg method. NES: Normalized Enrichment Score. **(F)** GSEA enrichment plot of DEGs found to be upregulated by COVID-19 monocytes compared to HIV-1 monocytes from the validation dataset, scored on DEGs found to be upregulated by COVID-19 monocytes compared to HIV-1 monocytes from analysis. *p*-values are adjusted with Benjamini-Hochberg method. NES: Normalized Enrichment Score.

SUPPLEMENTARY FIGURE S5 Differential abundance and gene expression of plasmablasts and B cells. **(A)** Box plots of plasmablast subset proportions in COVID-19 patients from validation dataset. Proportions are computed for each patient by dividing their number of plasmablasts by their total number of B cells + plasmablasts. *p*-values are computed with Wilcoxon signed-rank test with Holm-Bonferroni adjustment. **(B)** Box plots of the proportions of B cell and plasmablast subsets across sex. Proportions are computed for each patient by dividing their number of cells in each B cell/plasmablast subset by their total number of B cells + plasmablasts. *p*-values are computed with Wilcoxon signed-rank test. **(C)** Box plots of the proportions of B cell and plasmablast subsets across acutely and chronically infected HIV-1^+^ patients. Proportions are computed for each patient by dividing their number of cells in each B cell/plasmablast subset by their total number of B cells + plasmablasts. *p*-values are computed with Wilcoxon signed-rank test with Holm-Bonferroni adjustment. **(D)** Box plots of average gene expression for key plasmablast genes, plotted for plasmablasts. *p*-values are computed with Wilcoxon signed-rank test. **(E)** Box plots of average gene expression for B cell activation genes, plotted for B cells. *p*-values are computed with Wilcoxon signed-rank test.

SUPPLEMENTARY FIGURE S6 HIV and COVID-19 signature enrichment of T cell DEGs. **(A)** GSEA enrichment plot of DEGs found to be upregulated by HIV-1 T cells compared to COVID-19 T cells from the validation dataset, scored on DEGs found to be upregulated by HIV T cells compared to COVID-19 T cells from analysis. P-values are adjusted with Benjamini-Hochberg method. NES: Normalized Enrichment Score. **(B)** GSEA enrichment plot of DEGs found to be upregulated by COVID-19 T cells compared to HIV-1 T cells from the validation dataset, scored on DEGs found to be upregulated by COVID-19 T cells compared to HIV-1 T cells from analysis. *p*-values are adjusted with Benjamini-Hochberg method. NES: Normalized Enrichment Score.

SUPPLEMENTARY FIGURE S7 Comparison of IFN-I gene expression across cell types and disease. **(A)** Violin plots of the normalized gene expression of the three IFN-I signatures, split across and major cell populations. **(B)** Heatmaps of HIV-1 IFN-I gene expression, plotted for severe COVID-19 patients and HIV-1^+^ individuals in the validation dataset. Expression is scaled across columns. **(C)** Heatmaps of COVID-19 IFN-I gene expression, plotted for severe COVID-19 patients and HIV^+^ individuals in the validation dataset. Expression is scaled across columns.

SUPPLEMENTARY FIGURE S8 Expression of actin polymerization and TLR signature scores across cell subtypes. **(A)** Box plots of average actin polymerization module score across all cell subtypes, split by disease status. *p*-values are computed with Wilcoxon signed-rank test with Holm-Bonferroni adjustment. **(B)** Box plots of average TLR signaling module score across all cell subtypes, split by disease status. *p*-values are computed with Wilcoxon signed-rank test with Holm-Bonferroni adjustment.

SUPPLEMENTARY FIGURE S9 Expression of metabolic genes across disease. **(A)** Expression heatmaps of key mitophagy genes plotted in T cells from validation dataset. Expression is scaled across columns. Columns are clustered based on Euclidean distance using complete clustering. **(B)** Expression heatmaps of key Rho GTPase genes plotted in T cells from validation dataset. Expression is scaled across columns. Columns are clustered based on Euclidean distance using complete clustering. **(C)** Expression heatmaps of key mTOR genes plotted in T cells from validation dataset. Expression is scaled across columns. Columns are clustered based on Euclidean distance using complete clustering.

SUPPLEMENTARY FIGURE S10 Comparison of expression of stress-associated genes across treatment and IFN-associated genes across disease status. **(A)** Box plots of average expression of FOSB, JUND, and NEAT1 genes across COVID-19 patients and healthy donors. *p*-values are computed with Wilcoxon signed-rank test. **(B)** Box plots of average expression of FOSB, JUND, and NEAT1 genes across Azithromycin-treated and untreated COVID-19 patients. *p*-values are computed with Wilcoxon signed-rank test. **(C)** Expression heatmaps of HIV-1 IFN-I genes plotted across disease condition. Expression is scaled across columns. Columns are clustered based on Euclidean distance using complete clustering. **(D)** Expression heatmaps of HIV-1 IFN-I genes plotted across disease status. Expression is scaled across columns. Columns are clustered based on Euclidean distance using complete clustering.
