# Supplementary figures and images for: A single-cell atlas reveals shared and distinct immune responses and metabolic profiles in SARS-CoV-2 and HIV-1 infections
Source: Front Genet. 2023 Mar 13;14:1105673. doi: 10.3389/fgene.2023.1105673 (PMC10040851; doi:10.3389/fgene.2023.1105673)

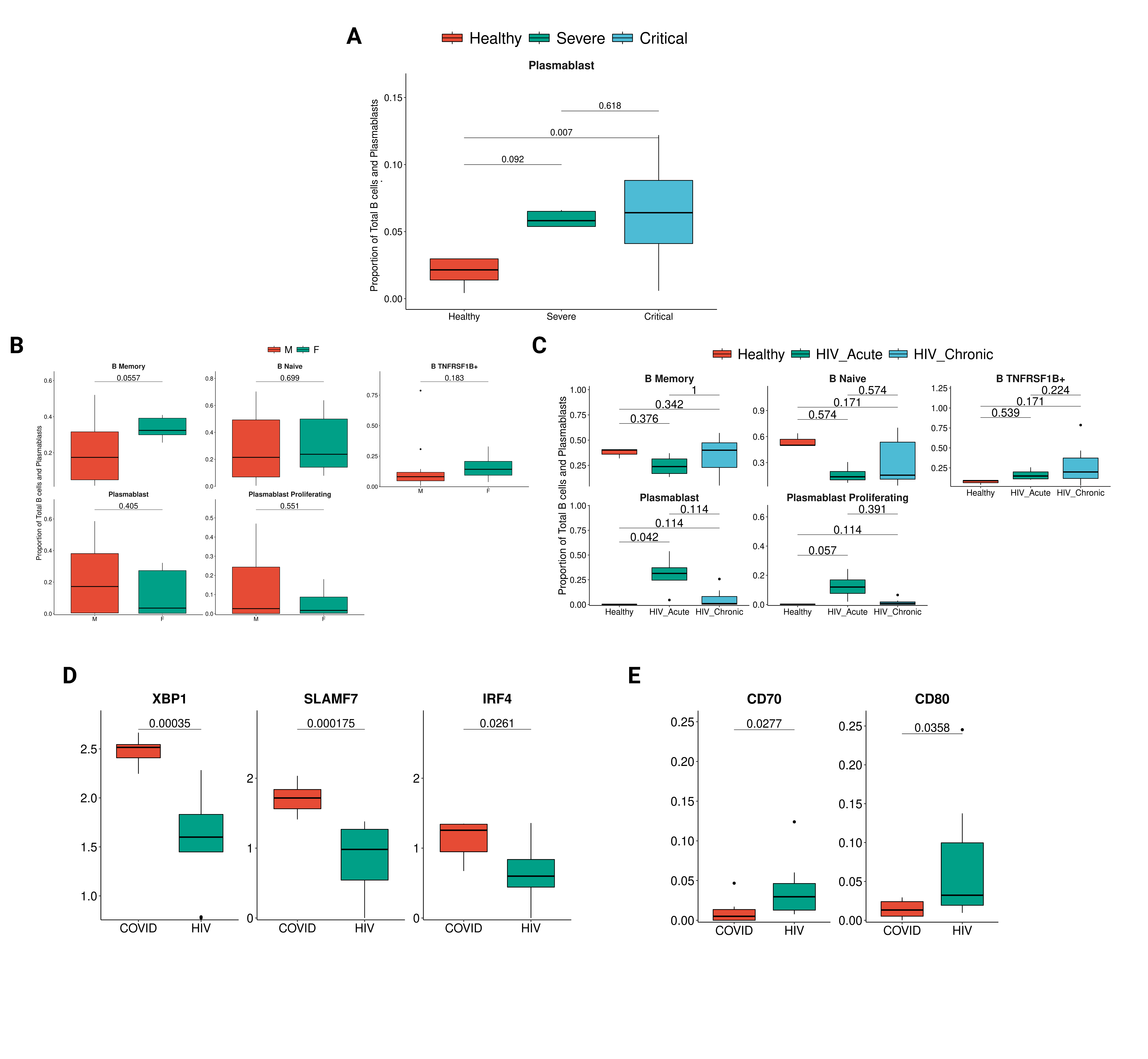

Supplement: Supplementary file 1 [file Image5.PNG]

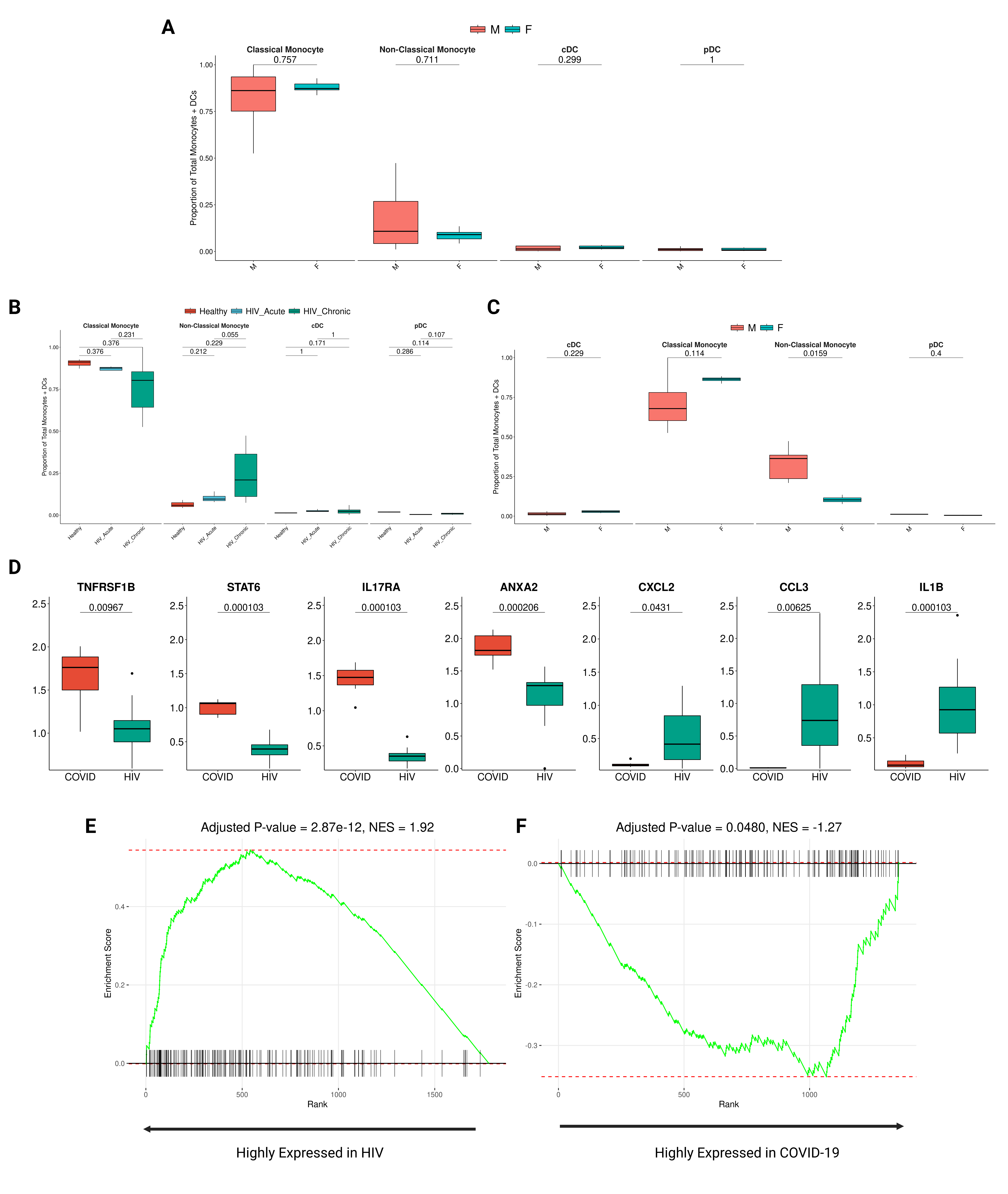

Supplement: Supplementary file 2 [file Image4.PNG]

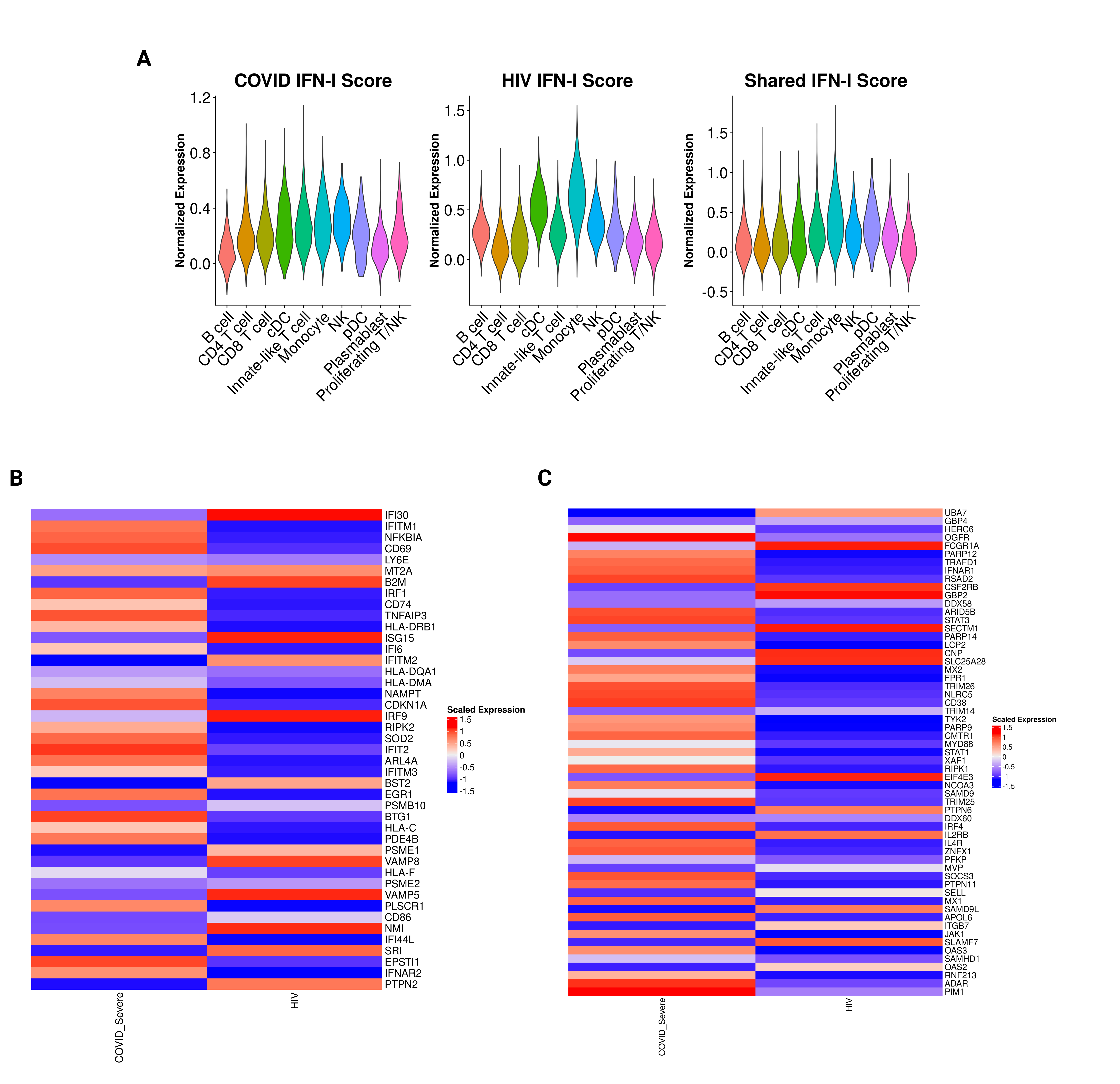

Supplement: Supplementary file 3 [file Image7.PNG]

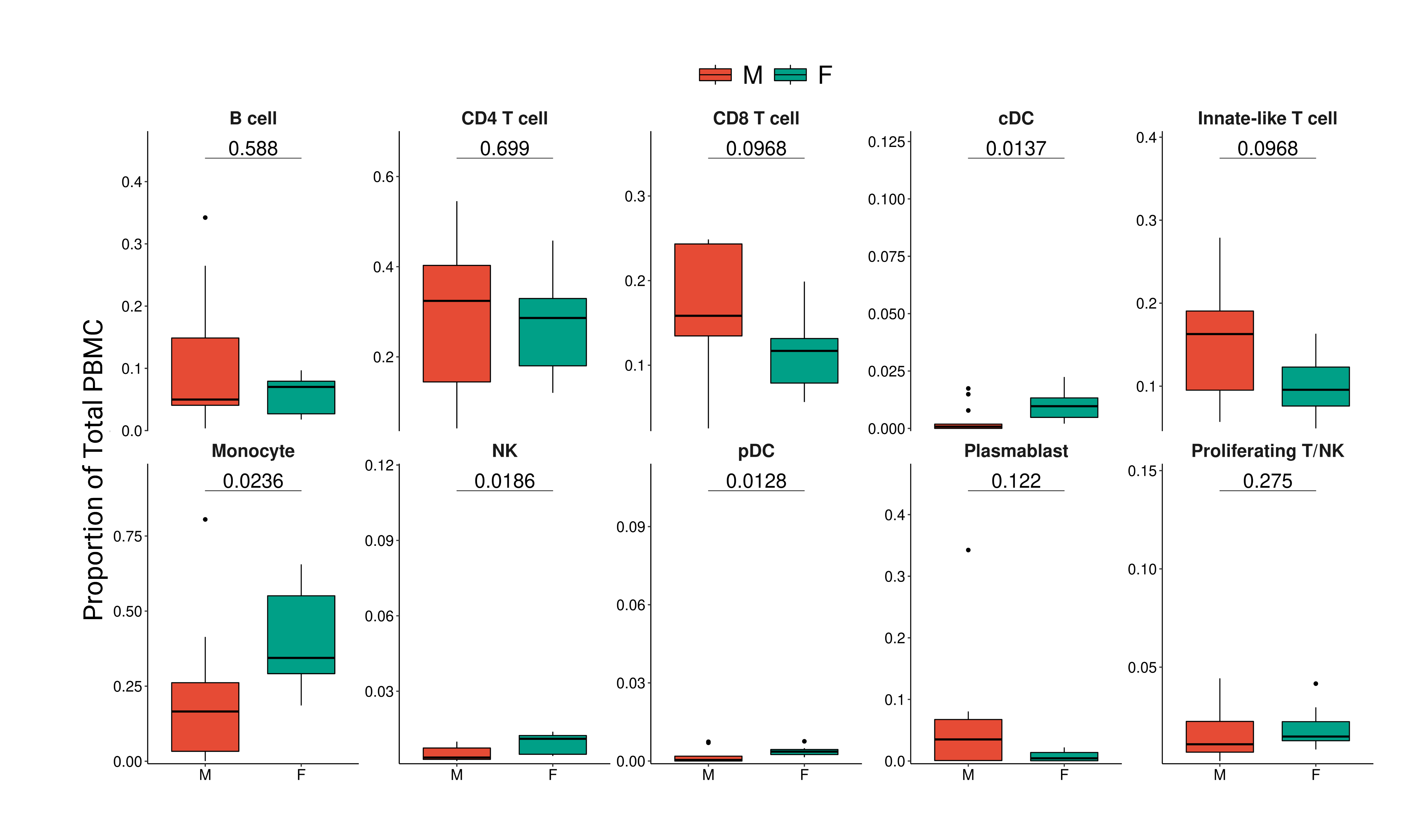

Supplement: Supplementary file 4 [file Image2.PNG]

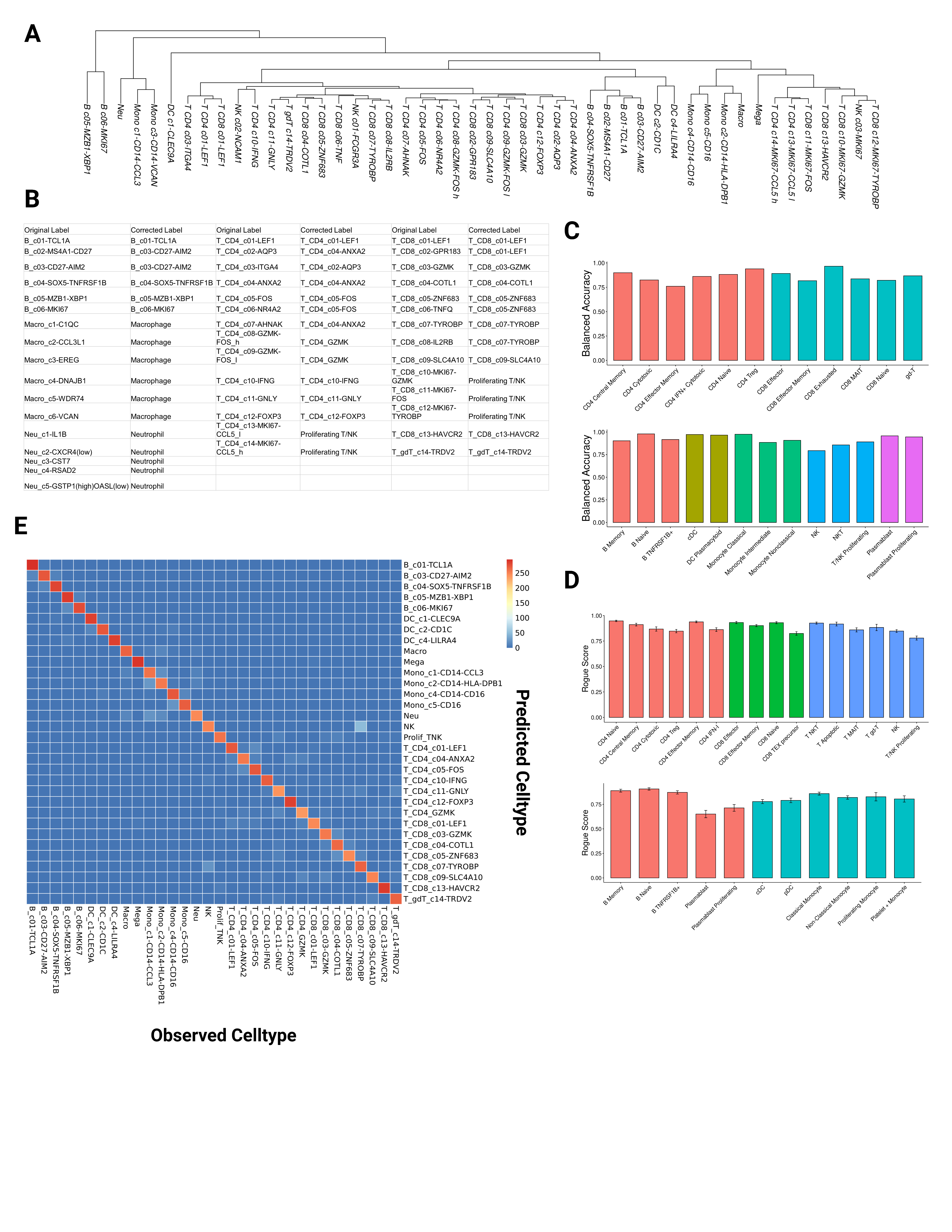

Supplement: Supplementary file 5 [file Image1.PNG]

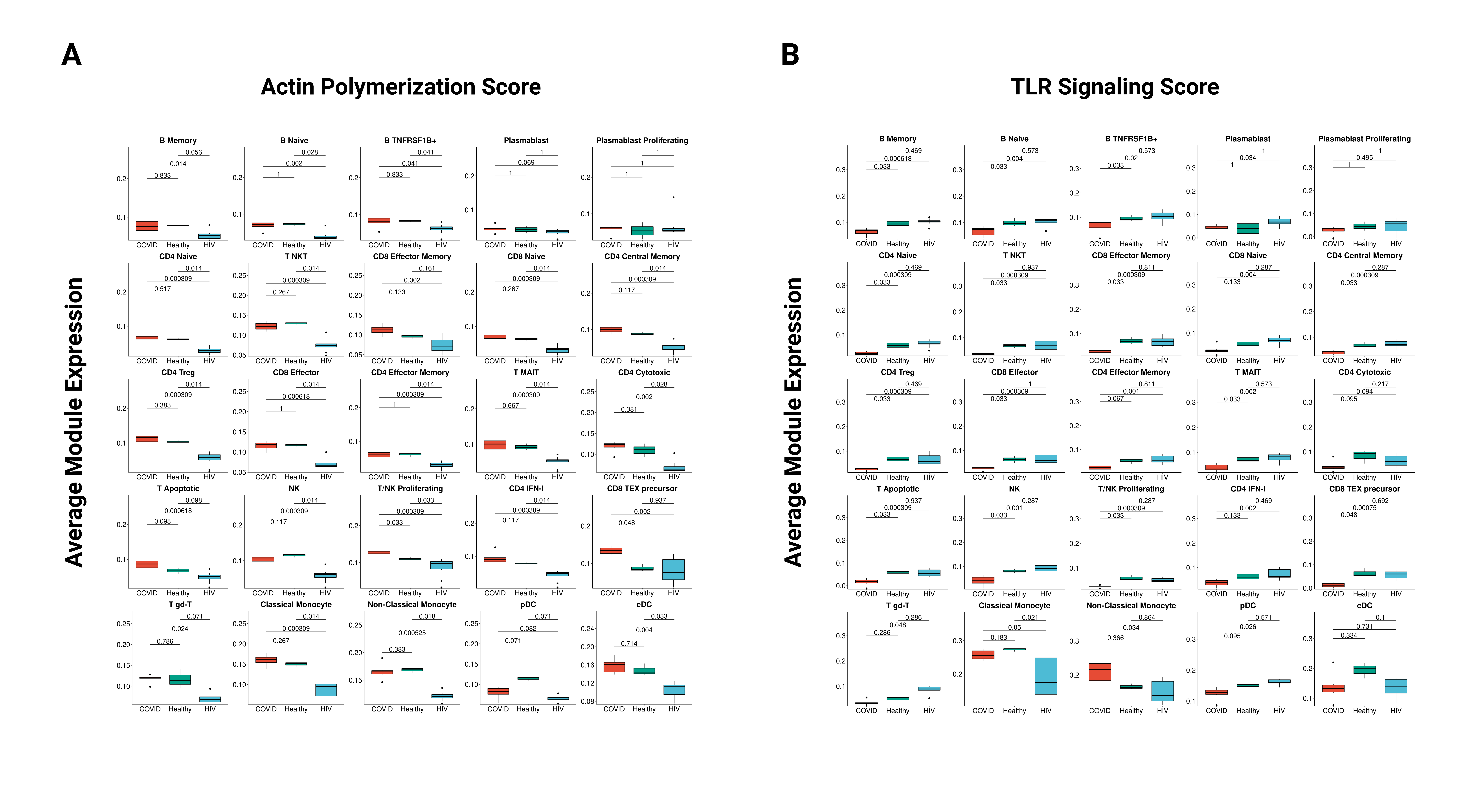

Supplement: Supplementary file 6 [file Image8.PNG]

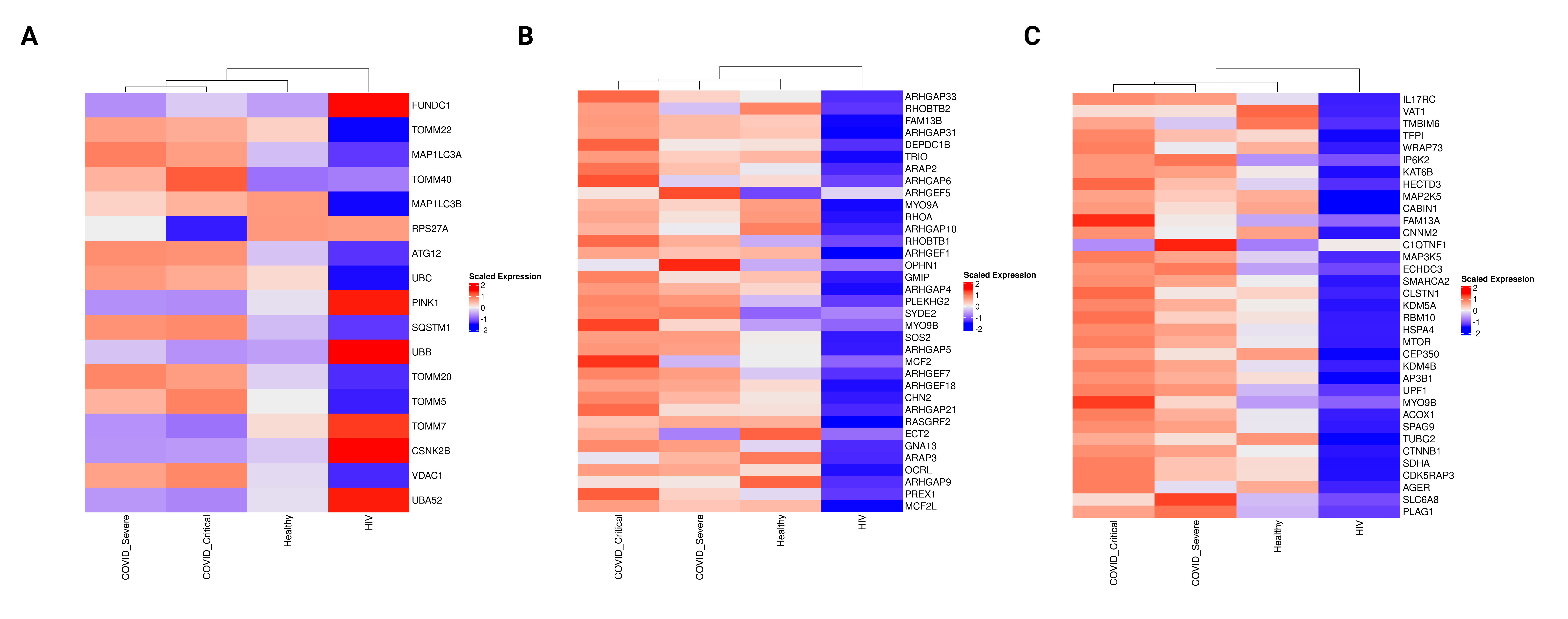

Supplement: Supplementary file 7 [file Image9.PNG]

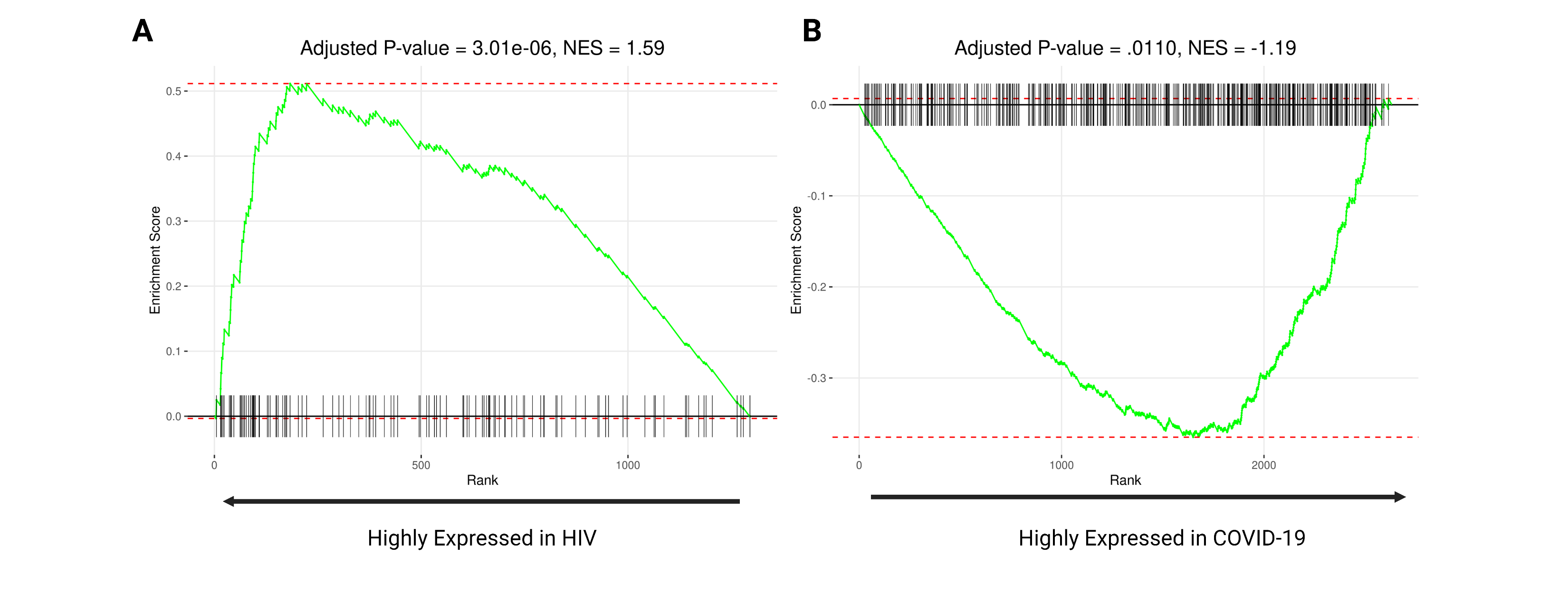

Supplement: Supplementary file 8 [file Image6.PNG]

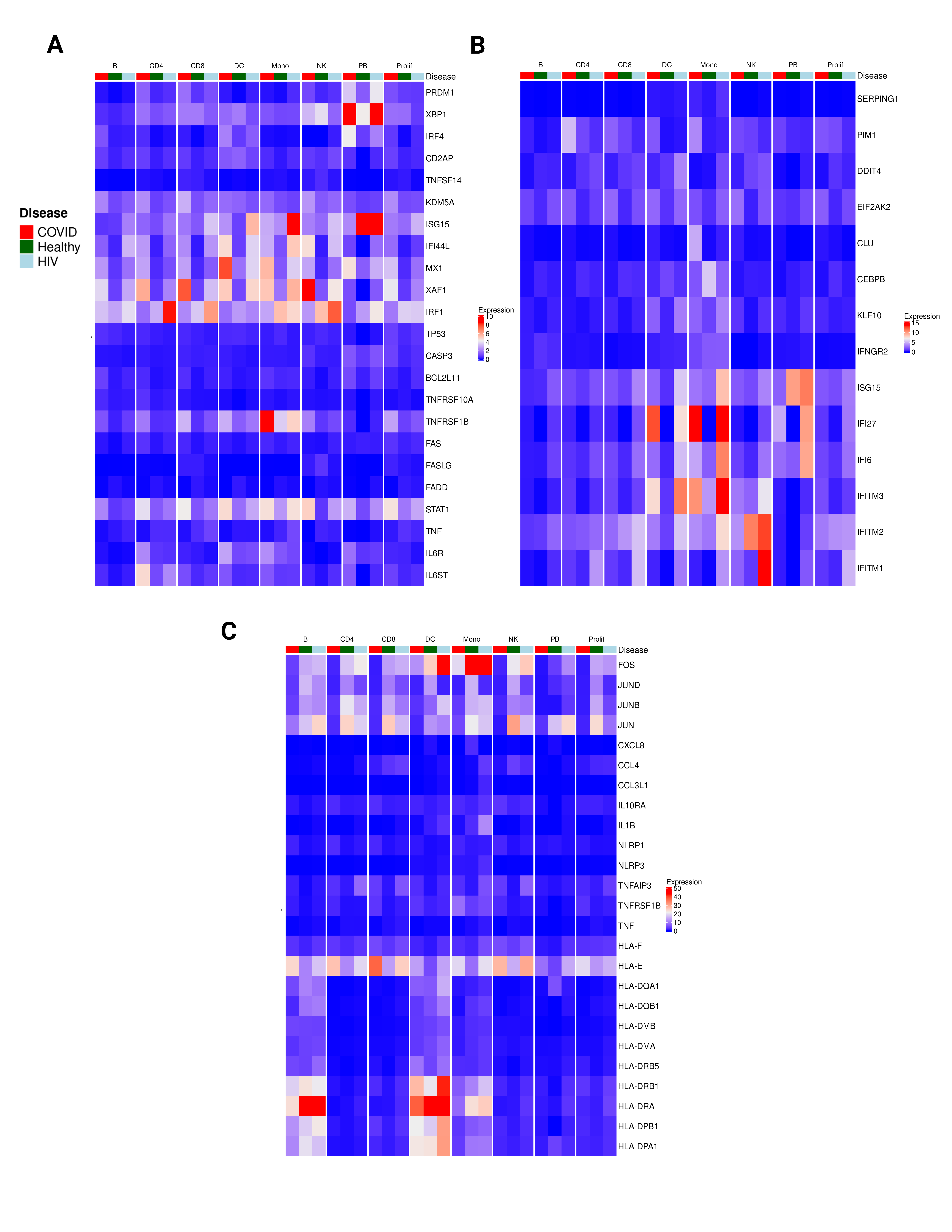

Supplement: Supplementary file 9 [file Image3.PNG]

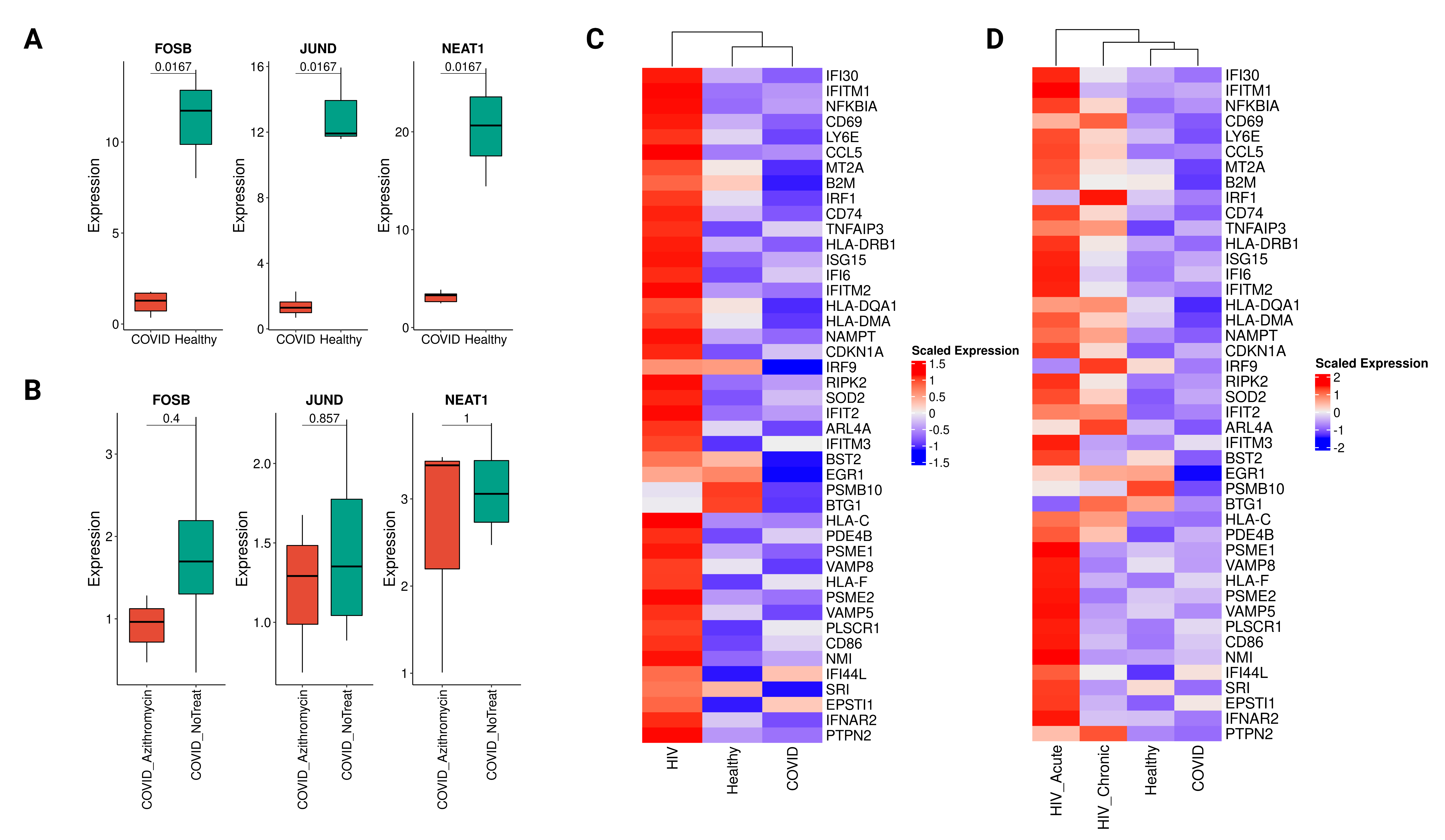

Supplement: Supplementary file 10 [file Image10.PNG]
